# Animal tracks and human footprints in prehistoric hunter-gatherer rock art of the Doro! nawas mountains (Namibia), analysed by present-day indigenous tracking experts

**DOI:** 10.1371/journal.pone.0289560

**Published:** 2023-09-13

**Authors:** Tilman Lenssen-Erz, Andreas Pastoors, Thorsten Uthmeier, Tsamgao Ciqae, /Ui Kxunta, Thui Thao

**Affiliations:** 1 Heinrich Barth Institute, Cologne, Germany; 2 Institute of Prehistory and Early History, Friedrich-Alexander-Universität Erlangen-Nürnberg (FAU), Erlangen, Germany; 3 Nyae Nyae Conservancy, Tsumkwe, Namibia; Griffith University, AUSTRALIA

## Abstract

Namibia is rich in hunter-gatherer rock art from the Later Stone Age (LSA); this is a tradition of which well-executed engravings of animal tracks in large numbers are characteristic. Research into rock art usually groups these motifs together with geometric signs; at best, therefore, it may provide summary lists of them. To date, the field has completely disregarded the fact that tracks and trackways are a rich medium of information for hunter-gatherers, alongside their deeper, culture-specific connotations. A recent research project, from which this article has emerged, has attempted to fill this research gap; it entailed indigenous tracking experts from the Kalahari analysing engraved animal tracks and human footprints in a rock art region in central Western Namibia, the Doro! nawas Mountains, which is the site of recently discovered rock art. The experts were able to define the species, sex, age group and exact leg of the specific animal or human depicted in more than 90% of the engravings they analysed (N = 513). Their work further demonstrates that the variety of fauna is much richer in engraved tracks than in depictions of animals in the same engraving tradition. The analyses reveal patterns that evidently arise from culturally determined preferences. The study represents further confirmation that indigenous knowledge, with its profound insights into a range of particular fields, has the capacity to considerably advance archaeological research.

## Introduction

Engravings of animal tracks and human footprints appear in numerous traditions of prehistoric rock art around the world. Examples include Patagonia, Argentina [1, 2]; Mamuno, Botswana [3]; Socotra Island, Yemen [4]; Limpopo, South Africa [5]; Galicia, Spain [6]; instances across Australia [7–9], whose Panaramitee rock art is of particular interest [10]; broad swathes of the US [11], one example being New Mexico [12] to the extent of a “Hoofprint Tradition of the Plains Indians” [11]; cases across northern Eurasia [13]; Nubia on the Nile [14]; Djado in Niger and Tibesti in Chad [15]; Zimbabwe [16]; Saudi Arabia [17]; and Upper Palaeolithic sites in Europe [18, 19]. The frequent occurrence of tracks as motifs for engravings contrasts with their virtual absence from paintings which, on a global scale, rather show hand prints. We call individual footprints—both animal and human—tracks, and a series of connected tracks a trackway. To ascertain the quantitative significance of track engravings in a relatively large region containing rock art, Richter and Vogelsang [20] compiled data on rock engravings collected by Scherz across Namibia. They found that more than 30% of the motifs are tracks, which do not feature at all in the paintings of that region, and that almost 5% show human feet and a few hands, in line with the proportion of hand signs in the region’s painted art [20]. The list compiled by Rudner and Rudner [21] in the 1960s shows that, according to the state of research of that time, track engravings occur at more sites in Namibia than in South Africa.

Research into prehistoric rock art usually categorises engravings of tracks together with geometric signs, leaving them badly under-researched despite their global ubiquity and broad chronological distribution [22]. Of the small number of studies that engage closely with the content and meaning of these motifs [e.g., 9, 23], many veer into esotericism [24]. Those studies directly incorporating indigenous knowledge are even fewer in number, and usually restrict themselves to referencing previously published stories and information [examples are 3, 25].

Imprints in soft ground left behind by moving organisms have served as important sources of information to people and populations throughout time, from the earliest hunter-gatherers [e.g., 26] to modern-day forensic investigators [e.g., 27]. Prehistoric hunter-gatherers arguably depended for their survival on their ability to draw as much information as possible from the tracks of animals and people. As much as prehistoric rock art itself is a global phenomenon, so is the occurrence of tracks within the spectrum of motifs. With this context in mind, this article rests on the working hypothesis that tracks in the rock art of the hunter-gatherers of the Later Stone Age (LSA, here referring to the last millennia BCE), in central western Namibia, have the capacity to convey information beyond simply identifying species. We argue that these engravings communicate additional information that only an accomplished track reader, such as an indigenous tracking expert, can interpret. To our knowledge, the study on which this article draws is the first to examine this hypothesis, whether specifically in Namibian rock art or on a global scale. To date, academics and amateurs alike have relied on their own personal knowledge when identifying species from tracks in rock art [16, 28]. Eastwood and Eastwood engaged a professional hunter to analyse engraved tracks, but their work did not go beyond identifying species [5]. This type of research has yet to lead to in-depth analysis of tracks in rock art, and accordingly has not yet gauged the potential for this type of motif to convey information in various forms. This article is our attempt to begin closing this research gap.

In recent years, archaeological research has increasingly begun to involve bearers of indigenous knowledge, particularly in ichnology, the science of tracks [29–31; see 32, 33 with specific relation to indigenous knowledge around prehistoric human tracks]. Some of this research has analysed, in much greater detail than had been accessible via the archaeological methods implemented hitherto, Pleistocene footprints preserved in caves in France that were in use during the Upper Palaeolithic [30, 31, 34]. We consider the undisputed skills and knowledge of indigenous hunters in tracking animals and humans equally successfully [18, 35–45; for pastoralists’ knowledge, cf. 46] as a methodological toolkit [47] with promising potential for other, related archaeological sources.

## Material

**Fig 1:**
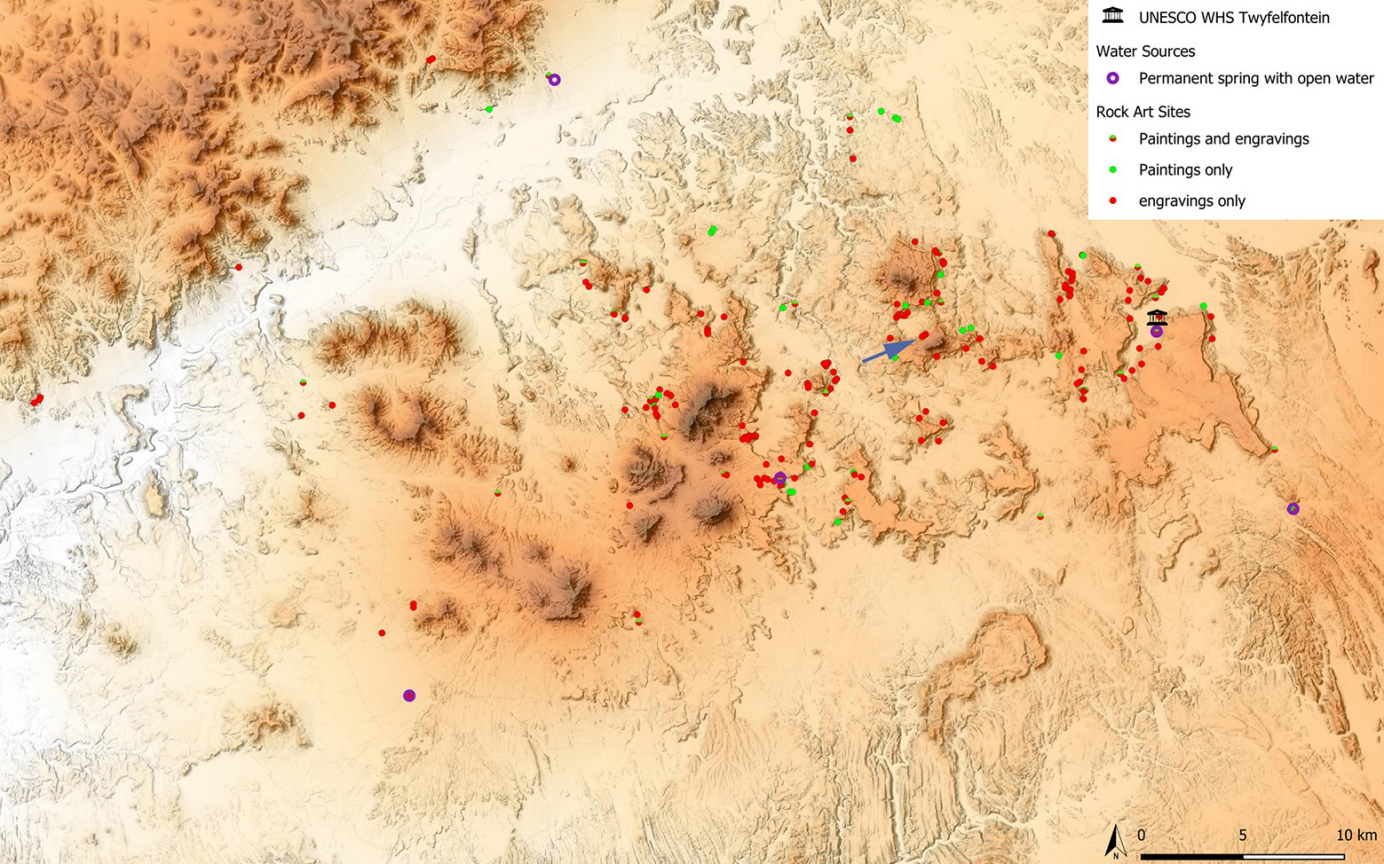
Map of the research area. Area covered by the research conducted by a team from Goethe University (Frankfurt); many of the sites are new discoveries. The arrow points to the two sites discussed in this article. Red = rock engravings; green = rock paintings; purple circles = permanent open water; black symbol = /Ui//aes-Twyfelfontein World Heritage Site (map by J. Behringer and P. Breunig).

**Fig 2:**
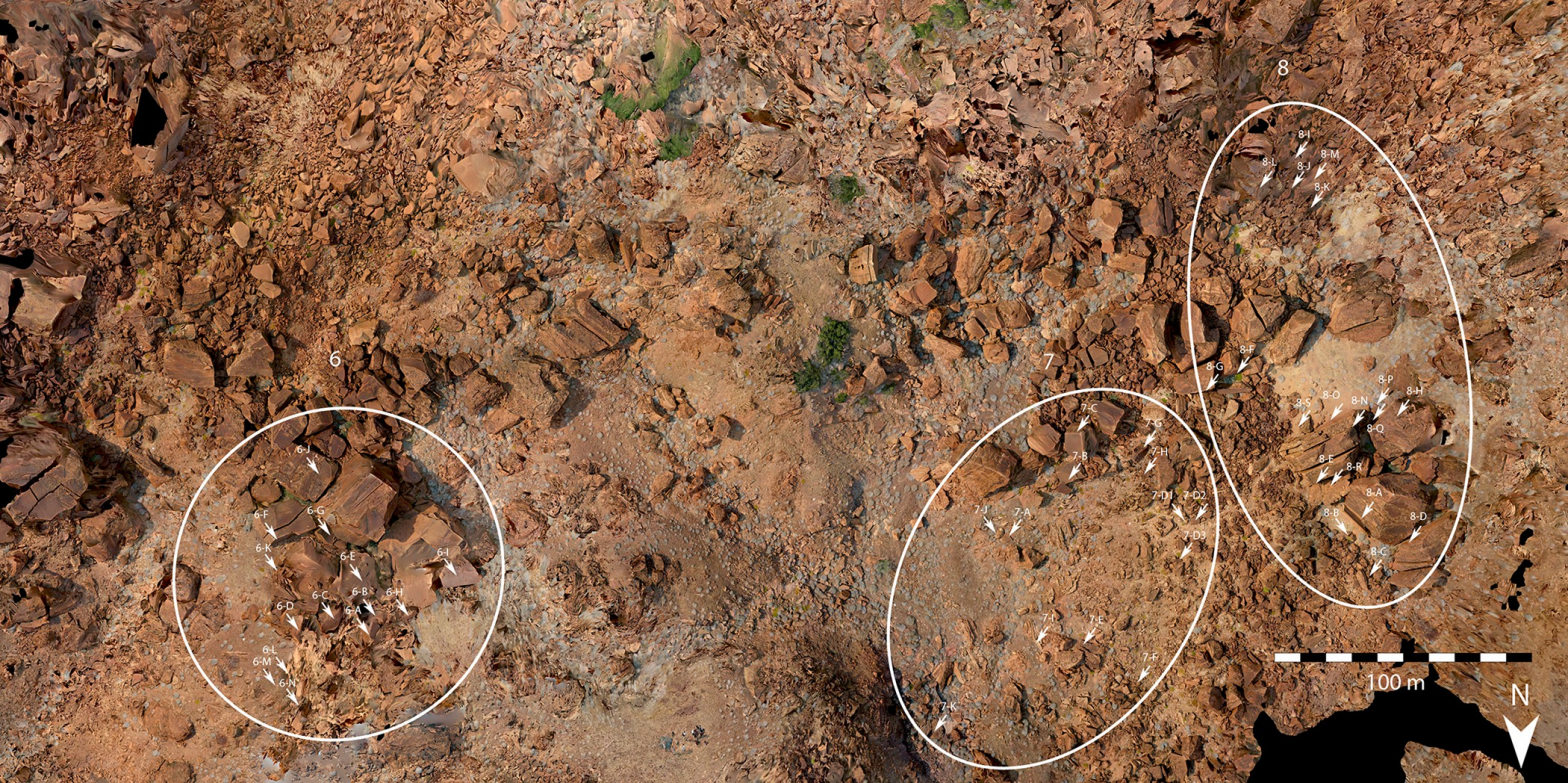
Layout of rock art sites RAS 6, RAS 7 and RAS 8. Aerial view of the layout of rock art sites RAS 6, RAS 7 and RAS 8 (photographs by O. Breunig; photogrammetry and map by P. Breunig).

The sites examined in this study are situated in the Doro! nawas mountains of Namibia. We chose them primarily for their abundance of track engravings on single panels. In an isolated, crater-like basin 11 km west of /Ui//aes-Twyfelfontein, two out of six rock art sites feature such panels. Sites RAS 6 and RAS 8 [48, 49 as numbered by Frankfurt Goethe University’s research project] are accumulations of sandstone boulders some 250 m from each other, both located on terraces of the basin’s steep south-eastern slope at roughly 800 m asl, while the bottom of the basin sits at 620 m asl (Figs 1 and 2).

Both sites are rich in archaeological finds of the pre-pottery LSA on several platforms; they are suitable for human settlement, but have little significant shelter. The two rocks at RAS 6 that were the subject of the investigation lie several metres apart with interspersed boulders between them, meaning no direct line of vision or hearing links them. Panel RAS 6-B North is the flat inclined upper part of a boulder; it sits some 1.5 m above the ground, so that the engravings cannot be seen undistorted from the bottom. Panel RAS 6-C is a vertical panel, but some of its engravings are on the upper side of the boulder, which is more than 2 m high; seeing them therefore requires the viewer to climb on top of the boulder, which is only possible from its back.

**Fig 3:**
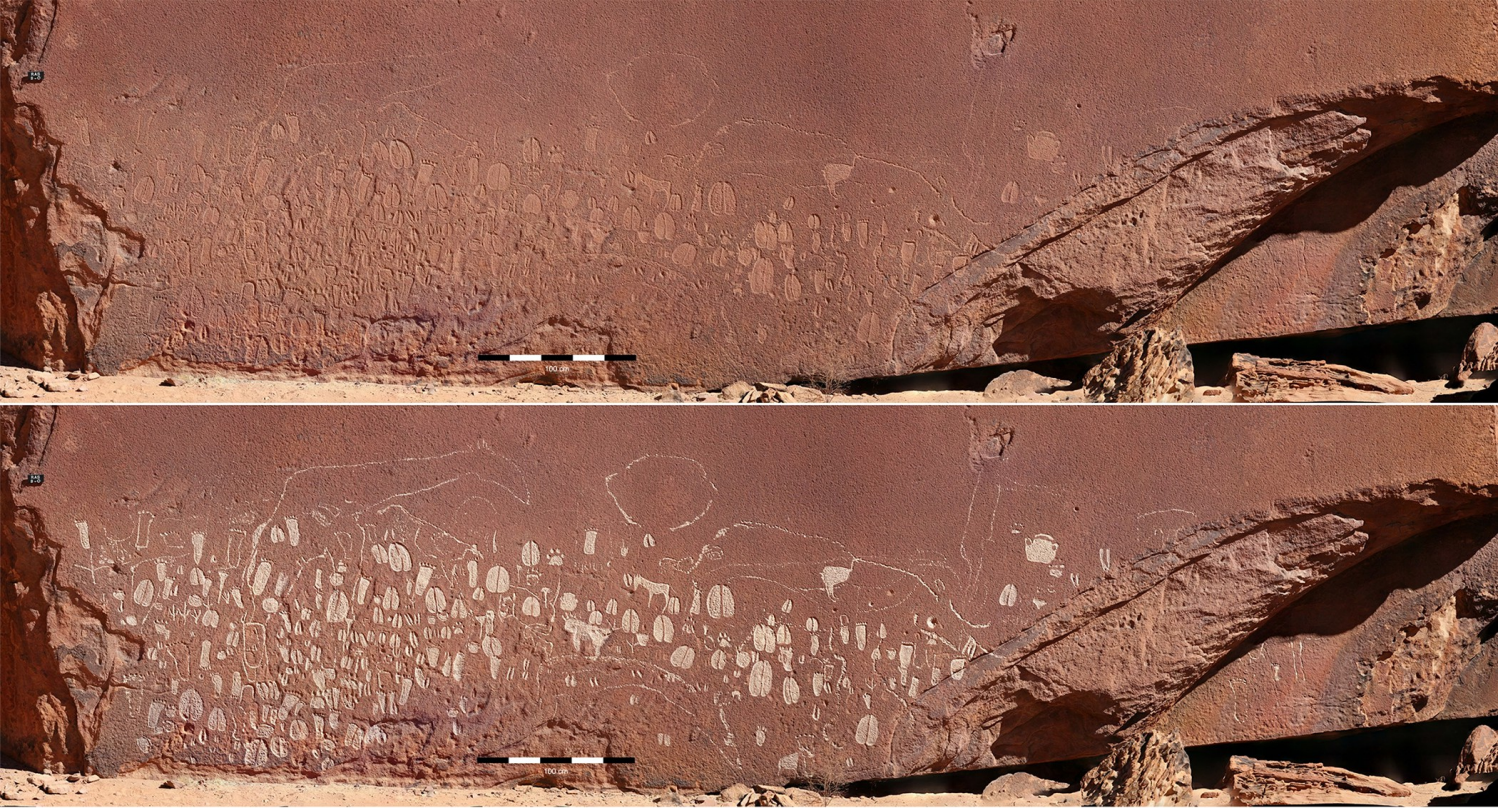
Main panel of the RAS 8 rock art site. RAS 8-O is the panel with the highest concentration of animal track engravings, amounting to almost 300 in number (images of engravings are digitally enhanced; photographs and artwork by P. Breunig).

**Fig 4:**
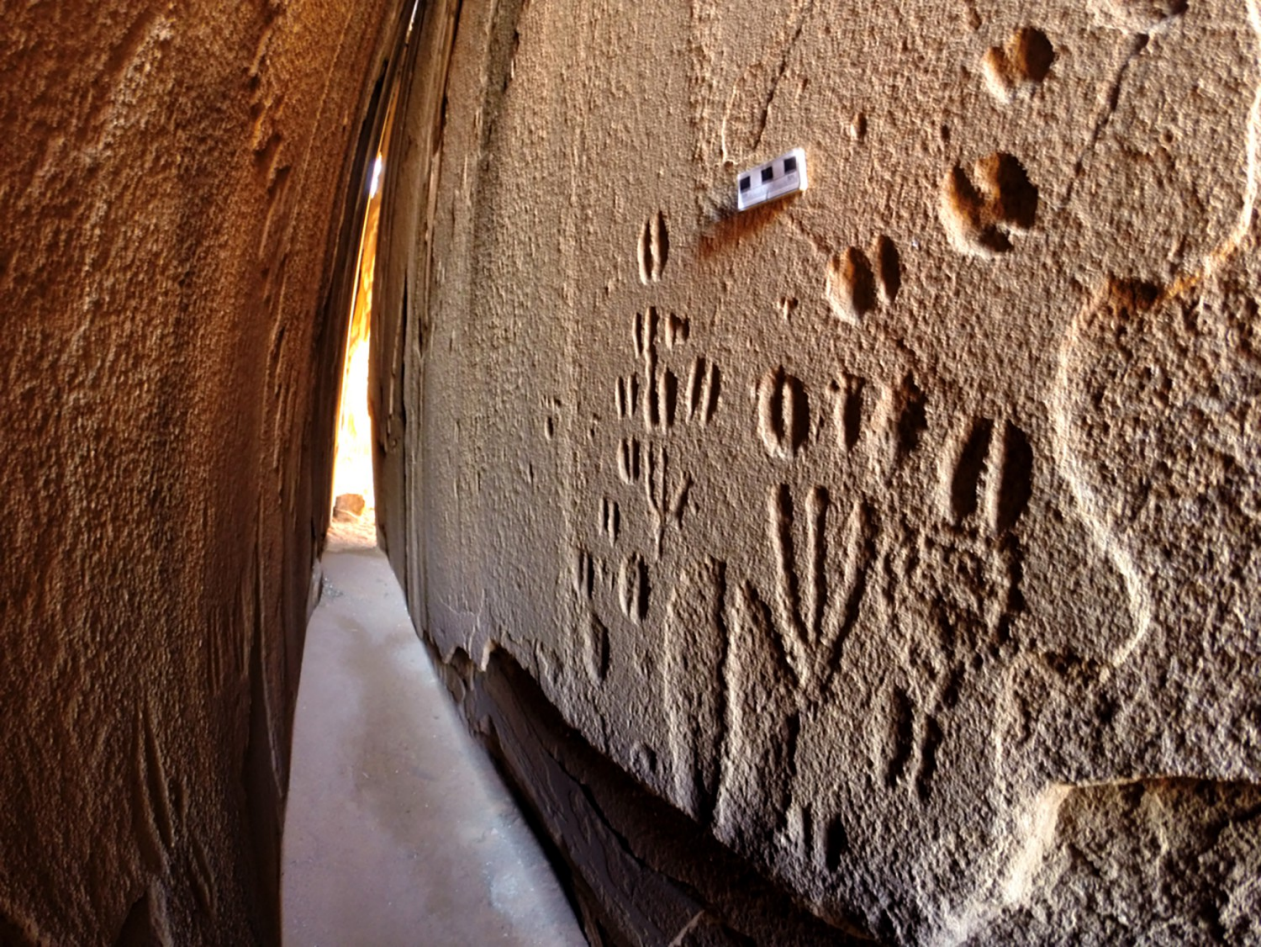
Hidden panel of the RAS 8 rock art site. The narrow corridor RAS 8-N, with engravings on both sides which extend as far inside as the lower exfoliated rock face; from the position of the photographer at the entrance, this is situated roughly three metres into the corridor (photograph: T. Lenssen-Erz).

The three panels at RAS 8 are all on the rim of a sizeable, secluded platform which provides a good view of the motifs–over 300 in number–of RAS 8-O (Fig 3) and those of RAS 8-H. RAS 8-N is an extraordinary configuration, virtually unique in Namibia and possibly beyond (Fig 4); the huge boulder bearing the engravings of RAS 8-O is estimated to be 5 m high, 10 m wide and 8 m thick. But along its width, parallel to the frontal panel RAS 8-O, the boulder is cracked in the middle, forming a straight crevice (Fig 4).

This crevice is only around 50 cm wide, with smooth, vertical walls that allow a person of average figure to pass through the boulder from one end to the other. Inside this very narrow corridor, which even at midday has only dim light, track engravings appear on both sides (RAS 8-N/NE and RAS 8-N/SW); there are no other recognisable motifs. The engravings begin directly at the southern entrance of the corridor and extend for another 3 m inside. Engravings also appear at the northern entrance to the corridor, but they are much fewer in number and do not extend into the corridor’s dim part. Many of the engravings inside the crevice are rather low on the wall; the corridor is too narrow to permit a person to bend down to see them, meaning that the adult or adolescent observer has to lie on the ground and pull themselves forward into the cleft. This art most likely being the work of adult or adolescent engravers, they must have taken a similar position to carry it out, without detriment, it appears, to the deployment of their skill, as the engravings show as much detail as those on the open outside walls. These motifs, then, are very well hidden, and two people at most can view them at any one time. This is inconsistent with virtually all other rock engraving sites, whose most frequently evident character is the openness of the display.

At both sites, RAS 6 and RAS 8, as in other sites in their environs, there are more panels that bear track and footprint engravings, but at lower concentrations; if we wished to expand the number of specimens for inclusion in a study, we would need to engage in a time-consuming process of surveying long stretches of this rocky terrain. The intense regional surveys conducted by the team from Goethe University, Frankfurt, revealed a total of 1824 track engravings at 60 sites, with a scattered distribution across 304 boulders (personal communication Manuela Fels, 22 Aug 2022).

**Fig 5:**
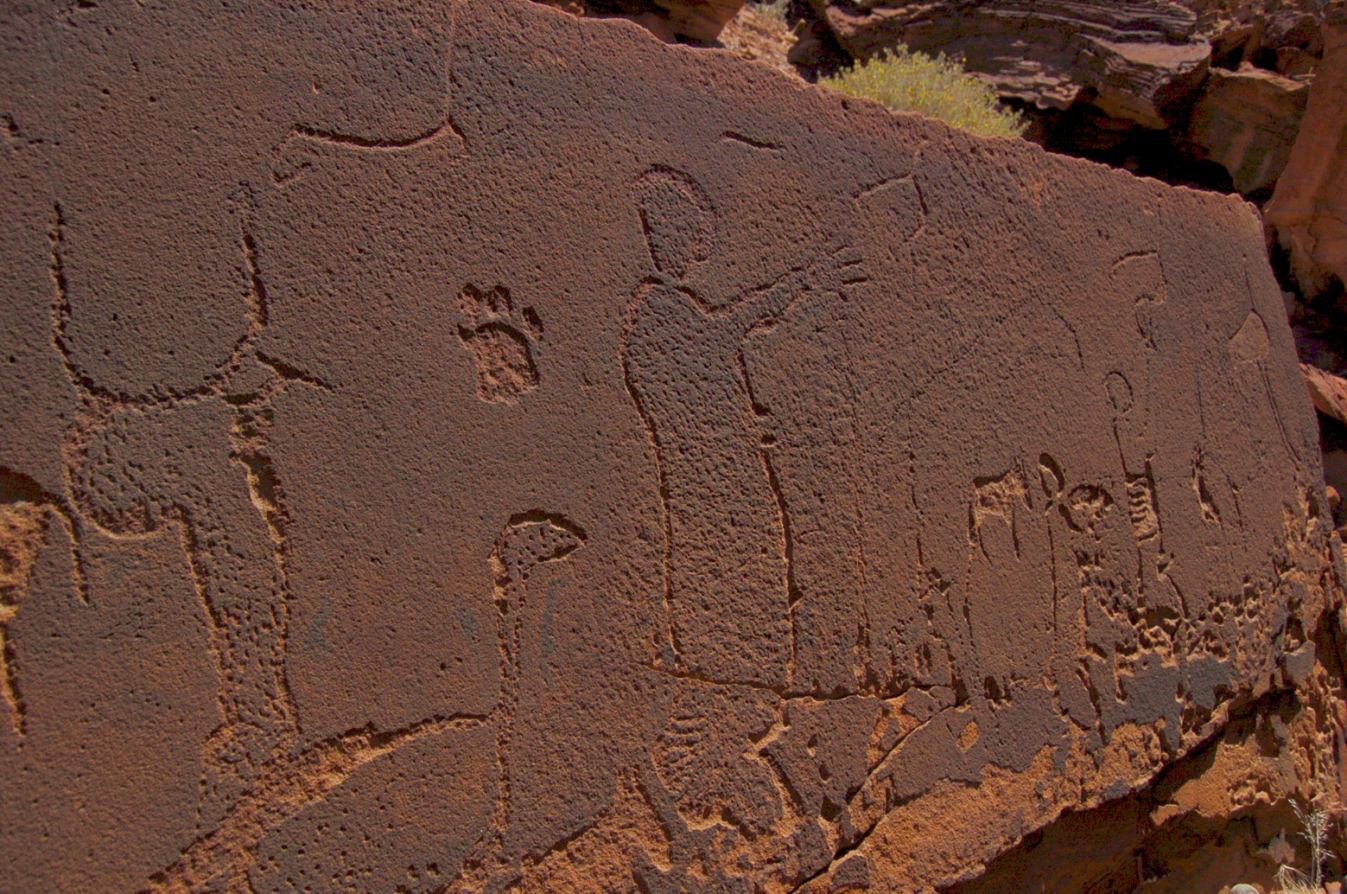
Large human figures at rock art site RAS 8. Panel RAS 8-A, showing, alongside the usual canon of animals, several human figures of up to 1 m in height, some men and at least one woman, all in the same position with their arm(s) extended to the right (photograph by T. Lenssen-Erz).

**Fig 6:**
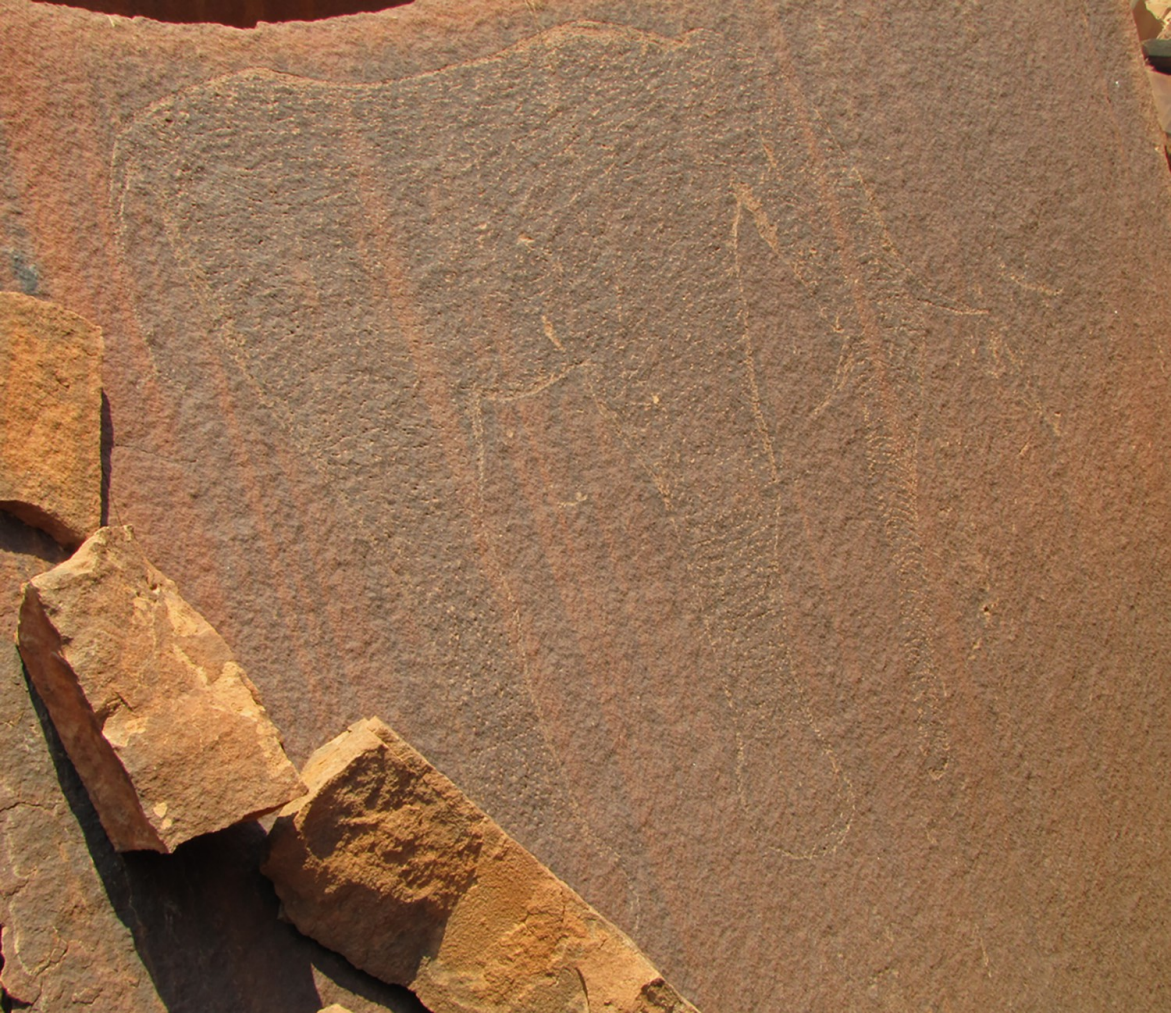
Large engraving of an elephant at rock art site RAS 8. Particularly large (height: 1.6 m) naturalistic elephant engraving on panel RAS 8 (photograph by T. Lenssen-Erz).

Doro! nawas is an area of about 50 x 30 km directly west of the World Heritage Site /Ui//aes-Twyfelfontein [50–54]. This proximity notwithstanding, discovery of the wealth of rock art in the area came about relatively recently, through the initiative of the amateur rock art enthusiasts Joe Walter and, secondarily, Uschi Kirchner [55]. Subsequently, a survey led by Peter Breunig of Goethe University endeavoured to create a comprehensive record of sites in this region; at the time of writing, mapping of more than 200 archaeological sites and features was complete [48, 49, 56] (Fig 1). /Ui//aes-Twyfelfontein is considered to be among the largest rock engraving sites in sub-Saharan Africa, with a rich variety of motifs. The sites at Doro! nawas located only a few kilometres to its west add considerably to this range; they include several large depictions of humans (Fig 5) and particularly large naturalistic engravings of elephants (Fig 6), neither of which occur at all at the World Heritage Site. It is the area’s wealth of animal track engravings that makes it an ideal subject for the present study.

Engravings are notoriously difficult to date. Iconographic considerations and stylistic similarities to the better-dated specimens permit us to date the rock engravings reported here only in a very broad frame; they presumably came into being between 5000 BP and roughly 1000 BP [53, referring to the art of /Ui-//aes-Twyfelfontein], or, as Breunig writes, in “‘the Holocene’, with a higher likelihood of late Holocene” [48]. The *terminus ante quem* established for the engravings of animal tracks found on a slab excavated in Zimbabwe’s Hwange National Park, having fallen off the shelter wall, is 2400 cal yrs BP, with hints at an even earlier genesis, prior to 3100 BP [57].

## Methods

For this study, we engaged three indigenous tracking experts, Tsamgao Ciqae, /Ui Kxunta and Thui Thao; they had previously worked on the Tracking in Caves project [30–32, 34], but also as professional trackers for commercial hunting. The research at the sites selected for this study, RAS 6 and RAS 8, took place from 18 to 20 September 2018. Additional information on the ethical, cultural, and scientific considerations relevant for our research but also specific to inclusivity in global research is included in the S1 Checklist.

### Photogrammetry models

**Fig 7:**
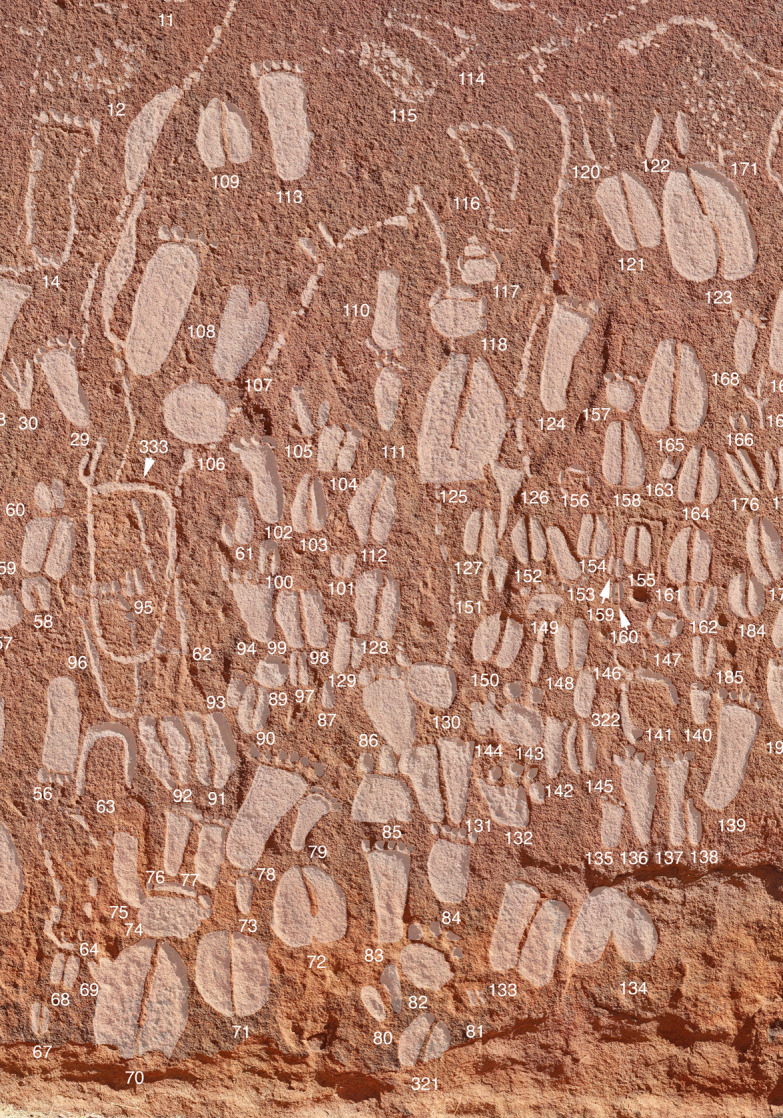
Documentation of rock art. Section of panel RAS 8-O, showing the individual sequential numbers for all engravings (digitally enhanced; height of section: c. 1.8 m; photograph and artwork by P. Breunig).

Photogrammetry models generated by the Goethe University team using the Structure from Motion method served as a basis for documenting the animal tracks and human footprints that occur in the engravings [49]. The software used to generate the models was Agisoft PhotoScan Professional. The orthophotographs produced in this way were digitally enhanced (Fig 3); we assigned an individual ID-number to every track identified (Fig 7).

### Intuitive morphometrics: Reading engravings of animal tracks and human footprints

**Fig 8:**
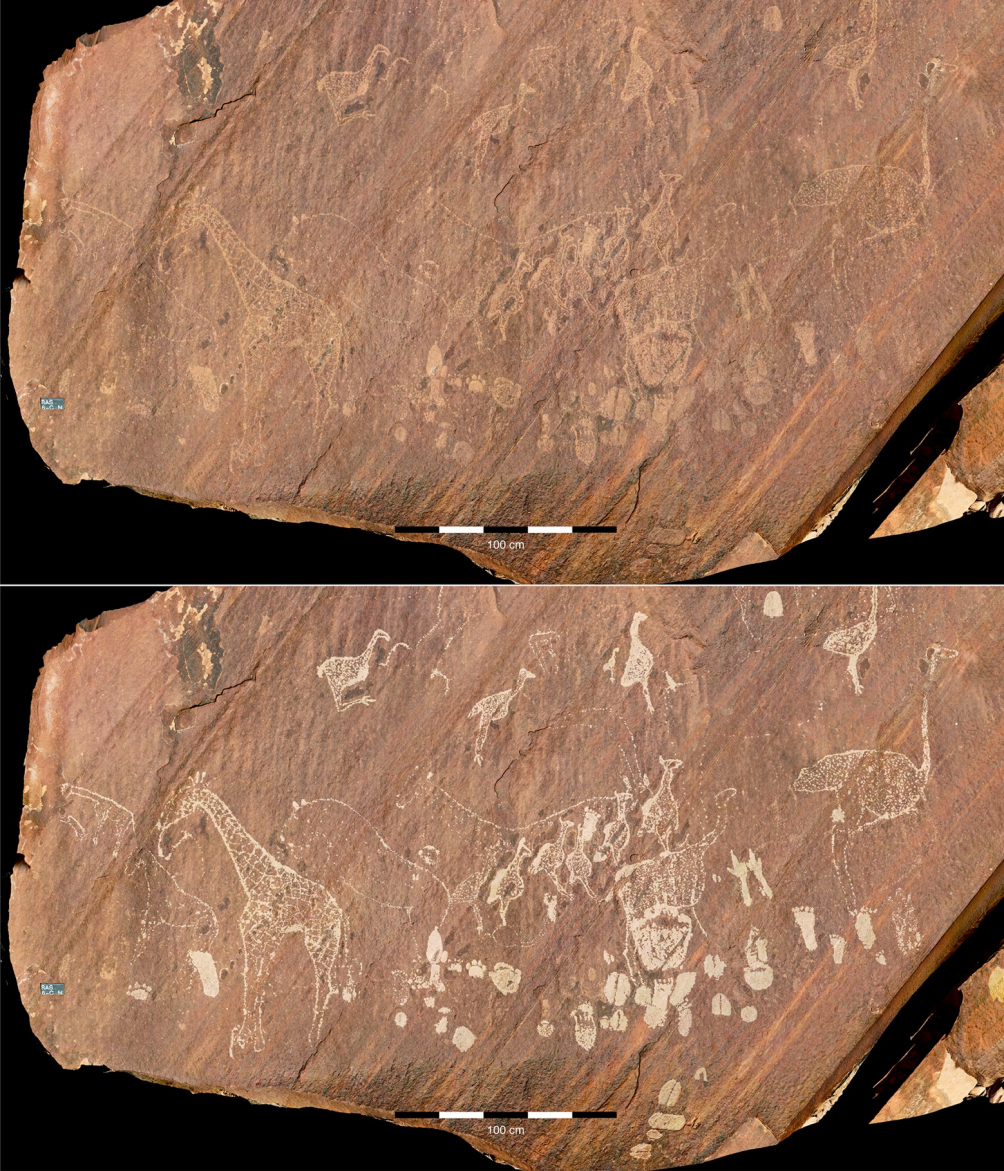
Exceptional panel from the RAS 6 rock art site. RAS 6-C is the most visible panel of RAS 6, featuring depictions of extraordinarily detailed giraffes and ostriches (above: engravings in their original condition; below: digitally enhanced images of the engravings; photographs and artwork by P. Breunig).

In general, we can consider the in-depth analysis of engraved animal tracks by indigenous tracking experts (Fig 8) to be methodologically closely related to the archaeological recording of animal motifs in rock art, which depends entirely on the researcher’s prior expertise [58] and rests fundamentally on the iconographic method as established by Panofsky [59].

In a procedure that we might call “intuitive morphometrics”, an observer attempts to match aspects of a work of art with phenomenological features she/he is capable of remembering from all animal species she/he has seen previously [60]. In most cases, this will be sufficient for the purposes of identifying species; sometimes it will be necessary to compare the artistic representation with the natural original [61, 62], and in a number of cases identification will be impossible, given the lack of direct information from the original artists [63, 64]. The success of this matching process, and the depth into which it can go, depend on the intensity and quantity of the specialist’s previous exposures to a feasibly large number of animals, with the consequence that the superior knowledge of a new track reader may lead to the re-interpretation of particular motifs [60, 63].

The Tracking in Caves project referenced above has standardised the process of recording the indigenous tracking experts’ workflow in reading prehistoric track engravings [30–32, 34]. The initial stage of this process entailed the compiling of lists with information on each individual track engraving examined. These lists documented details as follows:

- Site of the engraving, identified by the numbering system established in the research project of the Goethe University, Frankfurt [49].
- Track number, an ID-number designating each individual pictorial artefact examined and listed in the project.
- Species: the trackers identified all animals by their names in their mother language, after which Tsamgao Ciqae rendered their common English names.
- Age group: young/old, broadly distinguishing non-adults from adults; we identified distinct baby feet among the human footprint engravings.
- Sex: if it was possible to identify the subject’s sex, we recorded it as female or male.
- Limbs: which of the two–or four–feet is/are depicted.
- Side: where it was possible to identify which side of the body the extremity shown was on, we recorded it as left or right.
- Trackway: we noted here whether the track is part of a series of tracks of the same subject and to which other tracks it is connected, specifying them by their track numbers.
- Event identification (sequences or pairings): summary of traces and/or trackways of individual or several subjects in their temporal, spatial and content-related connections with one another, each track being identified by its ID number.
- Relative direction: the direction of the track on the rock wall, i.e. upwards/right/downwards/left.
- Preservation: the extent of the certainty to which track readers can identify an engraved track is subject to how well the engraving has endured, as well as to the care evidently taken in its execution and to the potential presence of superimpositions. We recorded the decline of preservation on a five-point scale from 1 = complete and clear to 5 = heavily damaged, barely recognisable.
- Execution: the investment of time, care and energy in the creation of an engraving, as deduced from its morphometrical features such as completeness and definition of outline; we recorded this on a five-point scale from 1 = clear and careful execution to 5 = very rough, incomplete and lacking precision.
- Superimposition: here, the scale of 1 to 5 expresses the area of a track whose identification is disrupted by a superimposition, from 1 = less than 20% to 5 = more than 80%. We additionally recorded the layer in a sequence of superimpositions in which a track occurred.
- Style: we assigned engravings to one of three categories, realistic, non-realistic or stylised.
- Design: this feature documented which of the two principal methods of rendering a track found use (in outline or full area).
- Remarks: an open field in the list for further comments of any kind.

We did not ascertain the sizes of the tracks on site, because the scaled orthophotographs enable the collection of this data *post hoc*. The indigenous tracking experts’ analyses were always entirely certain, unanimous and clear, irrespective of the actual size of a track representation which very often were not at a realistic scale.

We located the position of each track representation using scaled orthophotographs. Video recording of all work sequences of the tracking experts that took place will enable the checking and comparison of findings and the conduction of further linguistic research on the experts’ discussion of each track in their Ju/’hoan mother tongue. This data collection in the field produced a database (catalogue) containing the findings of this experiential, intuitive morphometric analysis conducted by the indigenous experts.

We can aid our understanding around the process of identifying a combination of track representations as a trackway, that is the interpretation of several tracks as a coherent event by considering the psychology of perception in general and Gestalt principles in particular [65]. Gestalt, a concept first formulated by Max Wertheimer [65], is “a unitary whole of varying degrees of detail, which, by virtue of its intrinsic articulation and structure, possesses coherence and consolidation and thus detaches itself as a closed unit from the surrounding field” [66, 67]. Research into Gestalt formation, which continues to this day, centres people’s perception and interpretation of grouped objects and of small entities within larger environments [68, 69]. What are referred to as Gestalt laws [70] or Gestalt principles are of particular relevance to the advertising industry [e.g., 71]; alongside psychology [e.g., 72], disciplines that have paid scholarly attention to them include computer science and mathematics [e.g., 73–75]. Some of the Gestalt principles or laws are figure-ground articulation, proximity, common fate, similarity, continuity, closure, past experience and good Gestalt [76]. All these principles are at work in the perception of single tracks and trackways and in the process of making sense of the complex information they provide.

### Statistical analysis

**Fig 9:**
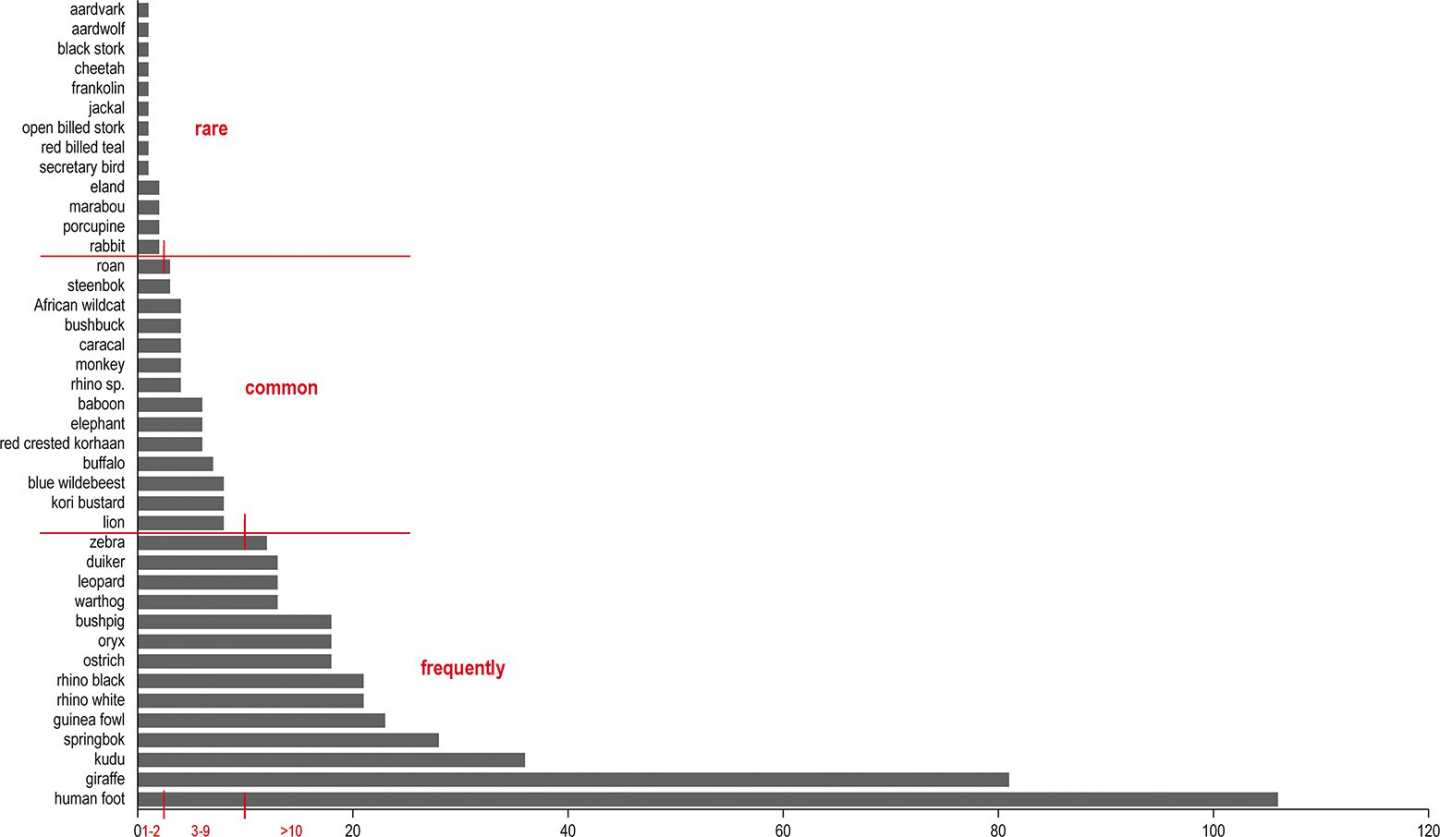
Absolute proportions of identified species. Graphical representation of the species identified by their tracks in the art found at selected Doro! nawas rock art sites. We omit tracks of animals with <10 occurrences from the analysis, due to the small sample size in these cases.

We used the statistical software PAST, version 4.11, to calculate descriptive statistics for the analysis of the data collected [77]. GeoRose version 0.5.1 created rose diagrams for visualising the relative direction of the animal tracks and human footprints [78]. To produce reliable results, we included in the analysis only those species identified by the tracking experts in ten or more track engravings (Fig 9).

## Results

The overall distribution of the engraved tracks and other motifs at the two sites RAS 6 and RAS 8 clearly shows that tracks play a significant role there (we note here that the Goethe University research team has documented further engravings of animals, humans and abstract signs at these sites). In most cases, there are marginally fewer depictions of tracks than of the other motifs at a site. By contrast, tracks are predominant on the RAS 6-B North panel. Panel RAS 8-O takes a special position within the rock art sites of the Doro! nawas mountains due to its extraordinary concentration of tracks, bearing more than half of all representations analysed in this study (Table 1).

**Table 1:** Numbers of motifs emerging from the analysis.

Panel	RAS 6-C	RAS 6-B North	RAS 8-H	RAS 8-N/NE	RAS 8-N/SW	RAS 8-O	TOTAL
Tracks	65	88	17	53	28	262	513
Other motifs	97	85	23	66	34	341	646

This breakdown of motifs shows the numbers of tracks and other motifs in evidence at the sites under investigation.

The sections that follow detail the findings of the analysis. We first discuss the depictions of animal tracks found and subsequently those of human footprints; in each of these sections, we include subsections covering–as far as the information is available–the species identified, the determination of the individuals’ age and sex, superimpositions, trackways and relative direction.

### Animal tracks

#### Species identified

Among the 513 tracks analysed in total, the experts identified 345 quadrupeds and 62 bird tracks (407 in total from 40 different species; Rhinoceros sp. is listed as a taxon, but not counted as a separate species). We divide these into a group of ’frequently’ depicted species (10 depictions or more), a second group of ‘less frequently’ depicted species (between 3 and 9 depictions), and a group of ’rarely’ depicted species (one or two specimens only) (Fig 9). The animal track engravings encompass 39 species, including herbivores, felines, other predators, birds and primates (Tables 2 and 12).

**Table 2:** Results of the age and sex determination process.

	Adult			Non-adult			All
Species	Female	Male	TOTAL	Female	Male	TOTAL	TOTAL
Aardvark	1	-	1	-	-	-	1
Aardwolf	-	1	1	-	-	-	1
African wildcat	-	4	4	-	-	-	4
Baboon	2	4	6	-	-	-	6
Black stork	-	1	1	-	-	-	1
Blue wildebeest	1	6	7	-	1	1	8
Buffalo	4	1	5	1	1	2	7
Bushbuck	2	2	4	-	-	-	4
Bushpig	3	6	9	2	7	9	18
Caracal	2	2	4	-	-	-	4
Cheetah	-	1	1	-	-	-	1
Duiker	1	6	7	2	4	6	13
Eland	-	2	2	-	-	-	2
Elephant	1	4	5	-	1	1	6
Francolin	-	1	1	-	-	-	1
Giraffe	23	31	54	19	8	27	81
Guinea fowl	12	7	19	3	1	4	23
Jackal	-	1	1	-	-	-	1
Kori bustard	3	5	8	-	-	-	8
Kudu	14	12	26	5	5	10	36
Leopard	6	4	10	3	-	3	13
Lion	1	6	7	-	1	1	8
Marabou	-	2	2	-	-	-	2
Monkey	1	2	3	1	-	1	4
Open-billed stork	-	1	1	-	-	-	1
Oryx	5	9	14	1	3	4	18
Ostrich	5	9	14	3	1	4	18
Porcupine	1	1	2	-	-	-	2
Rabbit	-	2	2	-	-	-	2
Red-billed teal	-	1	1	-	-	-	1
Red-crested korhaan	4	2	6	-	-	-	6
Rhino black	7	8	15	4	2	6	21
Rhino sp.	-	1	1	3	-	3	4
Rhino white	7	9	16	4	1	5	21
Roan antelope	-	3	3	-	-	-	3
Secretary bird	-	1	1	-	-	-	1
Springbok	9	15	24	4	-	4	28
Steenbok	2	1	3	-	-	-	3
Warthog	4	5	9	2	2	4	13
Zebra	1	8	9	1	2	3	12
TOTAL	122	187	309	58	40	98	407

Results of the process determining the age and sex of the animals depicted in the tracks investigated.

The first group of species comprises those ‘frequently’ depicted, that is, occurring in ten or more track engravings (Fig 9). In descending order of frequency, they are: giraffe (*Giraffa cameleopardalis*), kudu (*Tragelaphus* s*trepsiceros*), springbok (*Antidorcas marsupialis*), guinea fowl (*Numida meleagris*), white and black rhino (*Cerathotherium simum* and *Diceros bicornis*), ostrich (*Struthio camelus*), oryx/gemsbok (*Oryx gazella*), bushpig (*Potamochoerus larvatus*), warthog (*Phacochoerus africanus sundevalii*), leopard (*Panthera pardus*), duiker (*Sylvicapra grimmia*), and zebra (*Equus zebra*).

**Fig 10:**
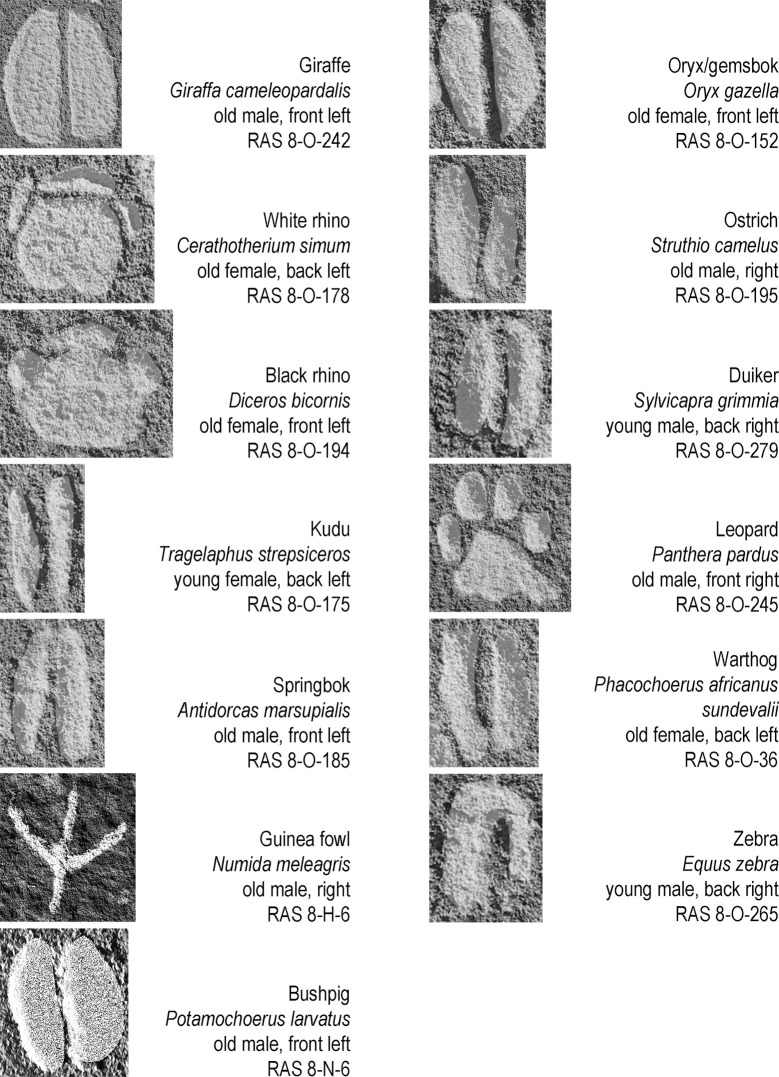
Examples of ‘frequently’ depicted animal tracks. The sequence in columns represents the order of number of occurrences. The scale of reproduction differs from picture to picture; all tracks are shown in upward direction (irrespective of their actual direction on the rock face); all tracks are digitally enhanced (excerpts from Fig 3, photographs and artwork by P. Breunig).

The experts identified black and white rhino in equal numbers (21 white rhino, 21 black rhino. They identified another four tracks at genus level only; we therefore classify these as Rhinoceros sp.) (Fig 10).

**Fig 11:**
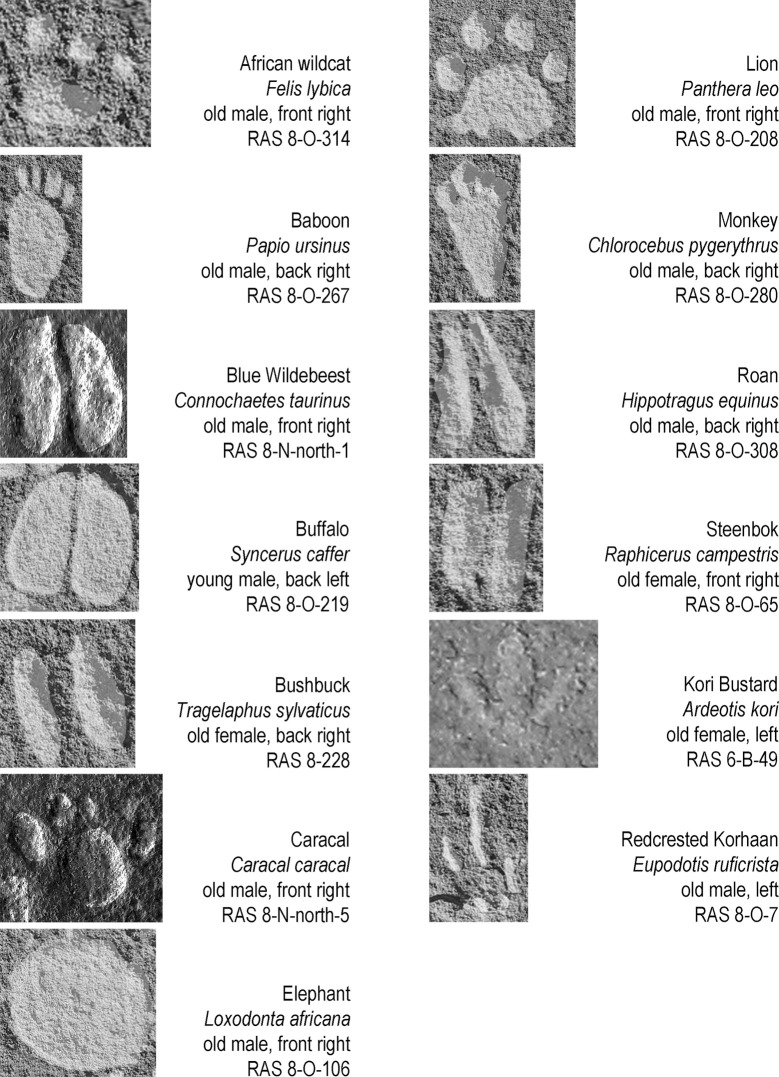
Examples of the ‘less frequently’ depicted animal tracks. The sequence in columns represents the order of number of occurrences. The scale of reproduction differs from picture to picture; all tracks are shown in upward direction (irrespective of their actual direction on the rock face); all tracks are digitally enhanced (excerpts from Fig 3, photographs and artwork by P. Breunig).

Among the ‘less frequently’ depicted group, featuring between three and nine tracks, were mammals as follows: African wildcat (*Felis lybica*), baboon (*Papio ursinus*), blue wildebeest (*Connochaetes taurinus*), buffalo (*Syncerus caffer*), bushbuck (*Tragelaphus sylvaticus*), caracal (*Caracal caracal*), elephant (*Loxodonta africana*), lion (*Panthera leo*), monkey (*Chlorocebus pygerythrus*), roan antelope (*Hippotragus equinus*) and steenbok (*Raphicerus campestris*). Two birds are also part of this group: kori bustard (*Ardeotis kori*) and red-crested korhaan (*Eupodotis ruficrista*) (Fig 11).

**Fig 12:**
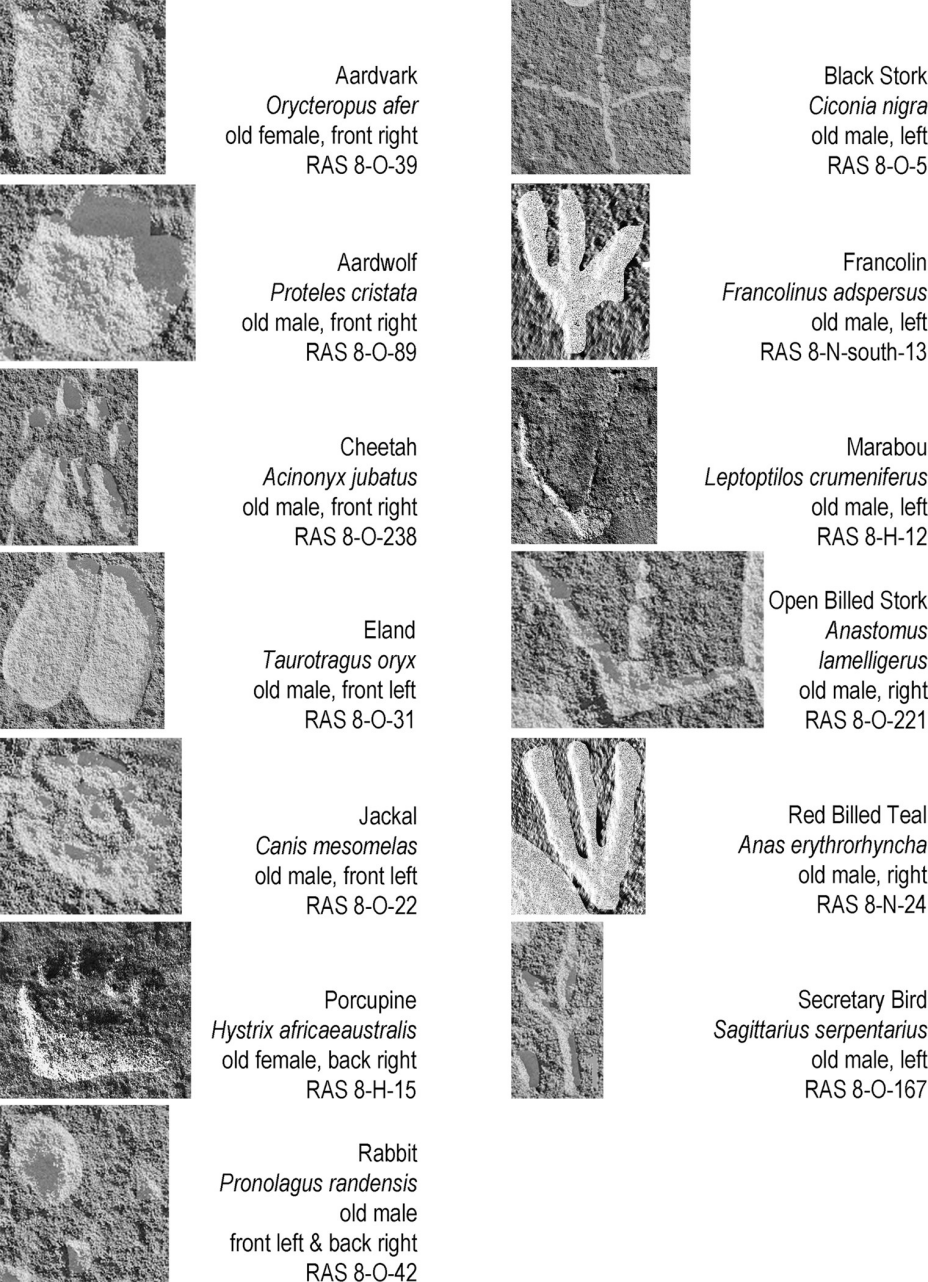
Examples of the ‘rarely’ depicted animal tracks. The sequence in columns represents the order of number of occurrences. The scale of reproduction differs from picture to picture; all tracks are shown in upward direction (irrespective of their actual direction on the rock face); all tracks are digitally enhanced (excerpts from Fig 3, photographs and artwork by P. Breunig).

Species whose tracks the experts identified only once or twice fall into the category of ‘rarely’ depicted animals. These are: aardvark (*Orycteropus afer*), aardwolf (*Proteles cristata*), cheetah (*Acinonyx jubatus*), eland (*Taurotragus oryx*), jackal (*Canis mesomelas*), porcupine (*Hystrix africaeaustralis*) and rabbit (*Pronolagus randensis*). Several species of bird also belong to this group: black stork (*Ciconia nigra*), francolin (*Francolinus adspersus*), marabou (*Leptoptilos crumeniferus*), open-billed stork (*Anastomus lamelligerus*), red-billed teal (*Anas erythrorhyncha*) and secretary bird (*Sagittarius serpentarius*) (Fig 12).

#### Determining the age and sex of animals represented by the tracks

Of the 407 animal tracks (345 quadrupeds and 62 birds) identified, a very clear predominance of adult animals is visible, with 309 tracks as compared to 98 from non-adults (Table 2). This evident preponderance of adult animals appears in almost all species, with three exceptions: bushpig and duiker, in which adult and non-adult tracks are approximately equal in number, and rhino sp., with non-adults predominating.

There are more tracks from males (N = 227) than from females (N = 180). Without going into too much detail at this point, we observe that these proportions are not the same across all species. Among buffalo, guinea fowl, kudu, leopard and red-crested korhaan, females are dominant. Differences in the distribution of the sexes across age groups are also evident in relation to some species; in the cases of giraffe, guinea fowl, ostrich, black rhino and white rhino, males outnumber females among adults and females outnumber males among non-adults. In general, we can observe that more tracks of young females than young males appear, in adult animal tracks, this distribution reverses with and more males than females occur.

#### Limbs/Legs and laterality

Regarding the limbs and legs associated with the tracks depicted, we present findings for quadruped and bird tracks separately, as birds, unlike quadrupeds, usually only leave tracks with their two legs.

In general, among quadrupeds, depictions of tracks from front legs appear significantly more frequently than those from hind legs, and tracks from left legs are depicted more often than those from right legs. However, if we break down the figures for laterality according to the limbs involved, it becomes apparent that the imbalance between left and right affects only the front leg, while the proportions of left and right are balanced in relation to hind legs. These results do not change when broken down by age group and sex of the animals depicted (Tables 3 and 4).

**Table 3:** Limb involved and laterality of engraved quadruped tracks: Analysis by age of animal depicted.

Principal characteristics	Hind leg				Front leg				TOTAL	
	Left leg		Right leg		Left leg		Right leg			
	N	%	N	%	N	%	N	%	N	%
Non-adult	15	4.3	14	4.1	39	11.3	22	6.4	90	26.1
Adult	32	9.3	30	8.7	112	32.5	81	23.5	255	73.9
TOTAL	47	13.6	44	12.8	151	43.8	103	29.9	345	100.0

Limb involved and laterality of engraved quadruped tracks, broken down by age group of the animals depicted.

**Table 4:** Limb involved and laterality of engraved quadruped tracks: Analysis by sex of animal depicted.

Principal characteristics	Hind leg				Front leg				TOTAL	
	Left leg		Right leg		Left leg		Right leg			
	N	%	N	%	N	%	N	%	N	%
Female	31	9	25	7,2	60	17,4	34	9,9	150	43,5
Male	16	4,6	19	5,5	91	26,3	69	20	195	56,5
TOTAL	47	13.6	44	12.8	151	43.8	103	29.9	345	100.0

Limb involved and laterality of engraved quadruped tracks, broken down by sex of the animals depicted

Tracks of birds show the same imbalance between left and right; breakdowns by age group and sex show a preponderance of the right leg among the small number of non-adult birds depicted (Tables 5 and 6).

**Table 5:** Leg involved and laterality of engraved bird tracks: Analysis by age of bird depicted.

Principal characteristics	Left leg		Right leg		TOTAL	
	N	%	N	%	N	%
Non-adult	3	4.8	5	8.1	8	12.9
Adult	34	54.8	20	32.3	54	87.1
TOTAL	37	59.7	25	40.3	62	100.0

Leg involved and laterality of engraved bird tracks, broken down by age group of birds depicted.

**Table 6:** Leg involved and laterality of engraved bird tracks: Analysis by sex of bird depicted.

Principal characteristics	Left leg		Right leg		TOTAL	
	N	%	N	%	N	%
Female	19	30,6	11	17,7	30	48,4
Male	18	29,0	14	22,6	32	51,6
TOTAL	37	59.7	25	40.3	62	100.0

Leg involved and laterality of engraved bird tracks, broken down by sex of the birds depicted.

#### Superimpositions

Of the 407 animal tracks, only 17 show superimpositions, affecting a total of ten species: blue wildebeest, duiker, giraffe (3x), jackal, lion (2x), oryx, black rhino, rhino sp., springbok (5x) and warthog. Due to the rareness of superimpositions it is impossible to derive a system or a rule according to which they were produced; it is evident that the overwhelming majority of track engraving procedures avoided producing superimpositions.

#### Trackways

Trackways, that is, trails of a certain individual over a particular depicted distance, are absolute exceptions among the rock art analysed; 368 of the 407 engravings show single tracks. The remaining animal tracks occur within a total of 18 short trackways mostly comprising two prints; the species featured are blue wildebeest, giraffe (2x), kori bustard (2x), leopard, marabou, oryx, ostrich, rabbit, red-crested korhaan, rhino sp., springbok and warthog. The only exception to these largely singular occurrences of trackways is the guinea fowl, of which three trackways of differing lengths exist, composed of two, three and four tracks respectively. The sample contains no depictions of events or scenes in which two or more individuals interact with one another.

#### Relative direction (direction of ‘walking’)

**Fig 13:**
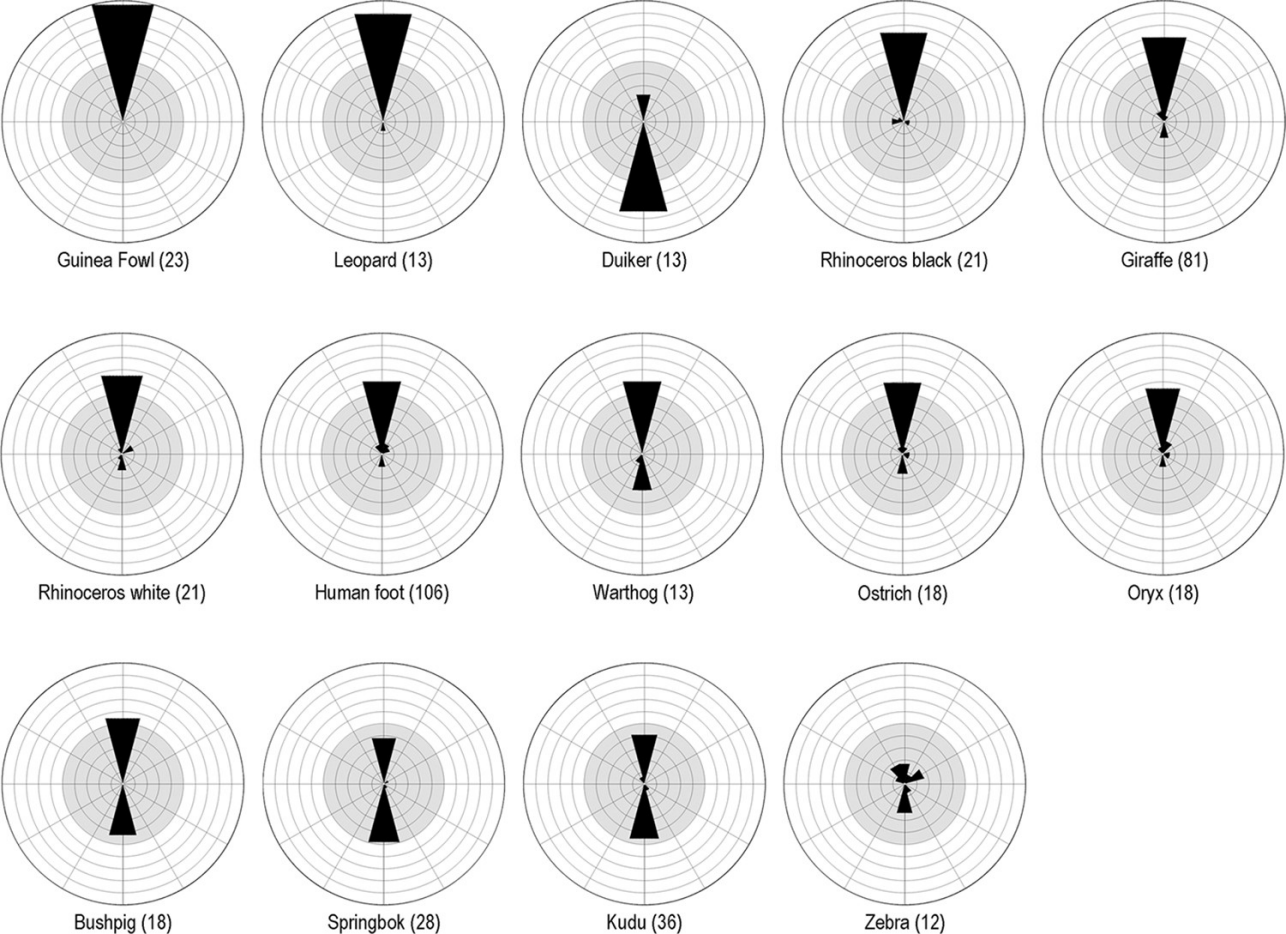
Directions of engraved tracks. The direction on the rock walls in which engravers depicted animal species’ tracks ‘walking’; this analysis is limited to those species of which ten or more engraved tracks were in evidence. We illustrate directions in the form of a wind rose diagram aligned in such a way that each direction shown corresponds to the viewer’s perspective.

Only 13 species (the two taxa of rhinoceros counted separately) make ten or more appearances with their tracks in the sample; in the interest of our findings’ reliability, we limited our analysis of relative direction of the tracks (that is, the direction in which they appear to be ‘walking’) to these species (Fig 9). As a rule, the predominant direction depicted is upward (vertically up the surface of the rock); some species, by contrast, were most typically depicted moving downwards. Of the 314 engravings showing tracks of the ‘frequently’ depicted animals, 215 point upwards and only 85 downwards. Engravers appear to have largely avoided a sideways direction; where it does occur, these tracks point mainly to the right and very rarely to the left (Fig 13; Table 7). Guinea fowl tracks point in only one direction; bushpig, duiker, giraffe, kudu, leopard and warthog tracks in two directions; oryx, ostrich, black rhino, white rhino and springbok in three directions; zebra tracks alone show all four principal directions (upwards, downwards, to the left and to the right).

**Table 7:** Direction of engraved tracks by species.

Species	Upwards		To right		Downwards		To left		TOTAL	
	N	%	N	%	N	%	N	%	N	%
Bushpig	10	55.6	-	-	8	44.4	-	-	18	100.0
Duiker	3	23.1	-	-	10	76.9	-	-	13	100.0
Giraffe	69	85.2	-	-	12	14.8	-	-	81	100.0
Guinea fowl	23	100.0	-	-	-	-	-	-	23	100.0
Kudu	17	47.2	-	-	19	52.8	-	-	36	100.0
Leopard	12	92.3	-	-	1	7.7	-	-	13	100.0
Oryx	13	72.2	-	-	4	22.2	1	5.6	18	100.0
Ostrich	13	72.2	2	11.1	3	16.7	-	-	18	100.0
Rhino, black	16	76.2	2	9.5	-	-	3	14.3	21	100.0
Rhino, white	15	71.4	2	9.5	4	19.0	-	-	21	100.0
Springbok	11	40.7	1	3.7	15	55.6	-	-	27	100.0
Warthog	8	61.5	-	-	5	38.5	-	-	13	100.0
Zebra	5	41.7	2	16.7	4	33.3	1	8.3	12	100.0
TOTAL	215	68.5	9	2.9	85	27.1	5	1.6	314	100.0

Direction of the engraved animal tracks, broken down by species.

Zebra is the only species for which it is impossible to identify a favoured direction of tracks. In all other species, regardless of the number of different directions depicted across the sample, one direction always predominates, accounting, as a rule, for at least 50% of the tracks of that species (Table 7).

We can distinguish two clusters of species by predominant direction; an upward direction features most frequently in tracks of giraffe, guinea fowl, leopard, oryx, ostrich, and black and white rhino, whereas duiker, kudu and springbok show a preponderance of downward-pointing tracks. Bushpig and warthog lie between the two categories because they have a preponderance of upwards-pointing tracks but in addition they appear with comparatively numerous downwards-pointing tracks.

**Fig 14:**
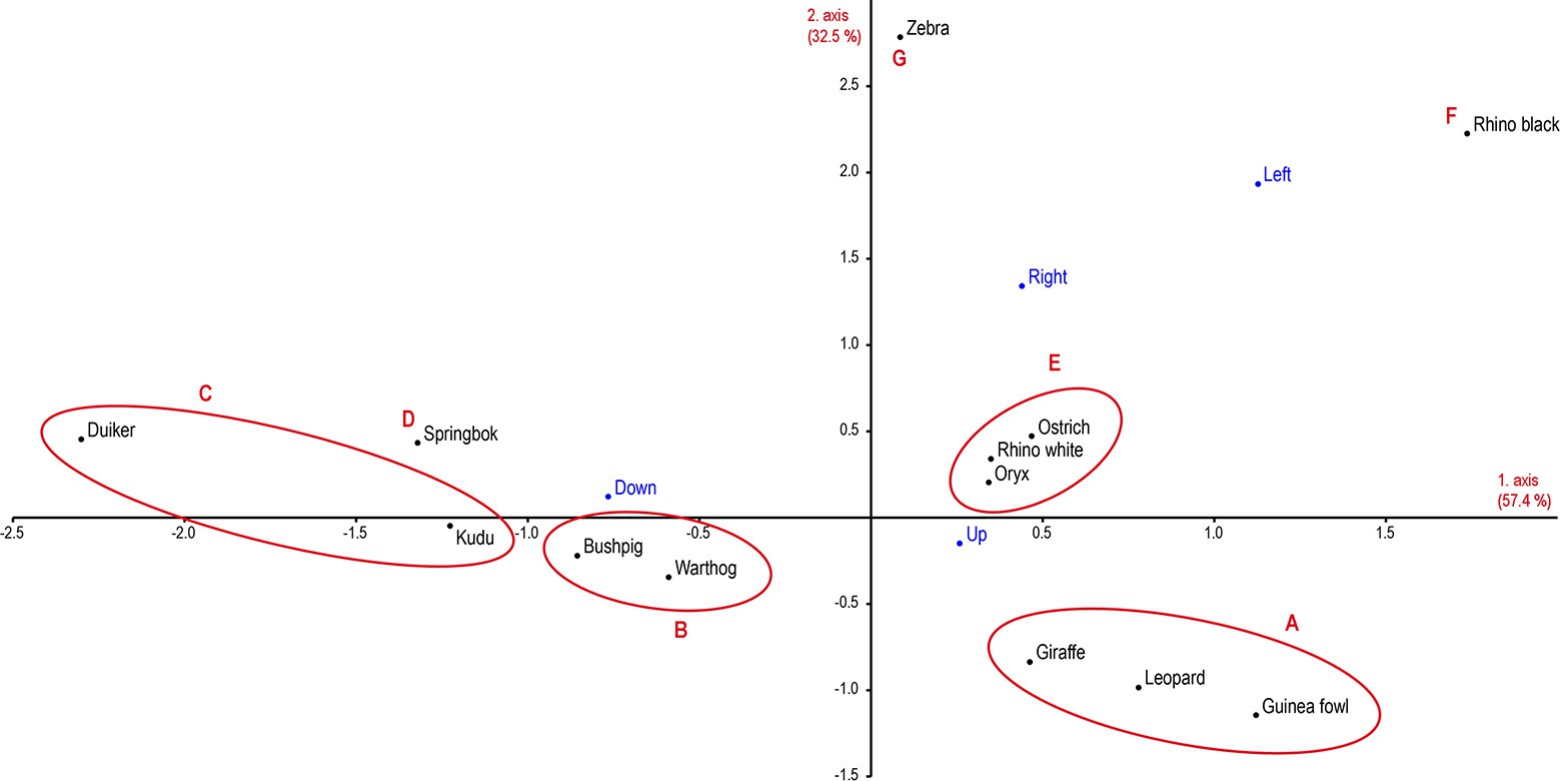
Correspondence analysis of directions of engraved tracks. Correspondence analysis of the directions of tracks engraved on the rock walls, including all animal species whose tracks occurred ten times or more (covering, on the first two axes, 89.9% of all cases).

We sought to refine our understanding of possible relationships between the direction of tracks and the species involved by conducting a correspondence analysis, in which each point in the two-dimensional space stands for the complete depiction profile (walking direction) of a species (Fig 14). In this case, the horizontal abscissa represents 57.4% and the vertical ordinate 32.5% of the original variations. Overall, the order shown in the two-dimensional representation of the data (Table 7) after the correspondence analysis (Fig 13) accounts for 89.8% of the variations; the information content is therefore very low, at around 10%. The sorting of the individual species by similarities in the numbers of directions depicted across the sample reveals seven clusters (A-G):

- Giraffe, leopard and guinea fowl, walking up- and downwards, with the upward direction predominant.
- Bushpig and warthog, walking up- and downwards, with equal proportions of up- and downward directions.
- Duiker and kudu, walking up- and downwards, with the downward direction predominant.
- Springbok, walking upwards, downwards and to the right, with the downward direction predominant.
- Ostrich, white rhino and oryx, walking upwards, downwards and to the right or to the left, with the upward direction predominant.
- Black rhino, walking upwards, to the right and to the left, with the upward direction predominant.
- Zebra, walking upwards, downwards, to the right and to the left, with equal proportions of up- and downward directions.

### Human footprints

About 20% of the track engravings investigated during this research are human footprints (106 of a total of 513).

#### Determining age and sex

We notice a preponderance of non-adult subjects among the human footprints; of 106 engravings of this type, the indigenous experts identified 88 as pertaining to non-adults, with only 15 deemed to stem from adult subjects. Three engravings of human footprints remained unassignable in this regard.

Differences in size and morphology permitted the similarly unambiguous determination of the sex of the human to whom the engraved footprint pertained; the analysis regarded 74 as male and 32 as female footprints. The breakdown by age group reveals a predominance of male subjects among adult and non-adult footprint engravings alike (Table 8).

**Table 8:** Results of the age and sex determination process for human footprints.

	Adult			Non-adult			No identification possible	All
Species	Female	Male	TOTAL	Female	Male	TOTAL		TOTAL
Human	4	11	15	28	60	88	3	106

Results of the age and sex determination process for the human footprints investigated in the study.

#### Laterality

The proportions of left and right human footprints are relatively balanced, with 50 representations of left and 56 of right feet. Breaking the findings down by age group and sex does not alter the picture (Tables 9 and 10).

**Table 9:** Laterality of engraved human footprints: Analysis by age.

Principal characteristics	Left leg		Right leg		TOTAL	
	N	%	N	%	N	%
Non-adult	40	38.8	48	46.6	88	85.4
Adult	8	7.8	7	6.8	15	14.6
TOTAL	48	46.6	55	53.4	103	100.0

Laterality of the engraved human tracks, broken down by age group.

**Table 10:** Laterality of engraved human footprints: Analysis by sex.

Principal characteristics	Left leg		Right leg		TOTAL	
	N	%	N	%	N	%
Female	14	13.2	18	17.0	32	30.2
Male	36	34.0	38	35.8	74	69.8
TOTAL	50	47.2	56	52.8	106	100.0

Laterality of the engraved human tracks, broken down by sex as determined by the analysis.

#### Superimpositions

Of a total of 106 human footprints, only ten show superimpositions; six of these instances involve other human footprints and four involve animal tracks from four different species (giraffe, lion, duiker and warthog).

#### Trackways

Of all human footprints analysed, only two pairs appear in two coherent sequences (thus forming the shortest possible trackway), among them two of the three sandals (i.e., unmistakable human footprints not showing toes, like the sole of a shoe) that form part of this body of art. In one place (RAS- 8-O), the left foot of a boy (no. 77) is depicted alongside and parallel to the left foot of a girl (no. 76); a Gestalt-influenced analysis would associate these two footprints with each other.

#### Relative direction of footprints

**Fig 15:**
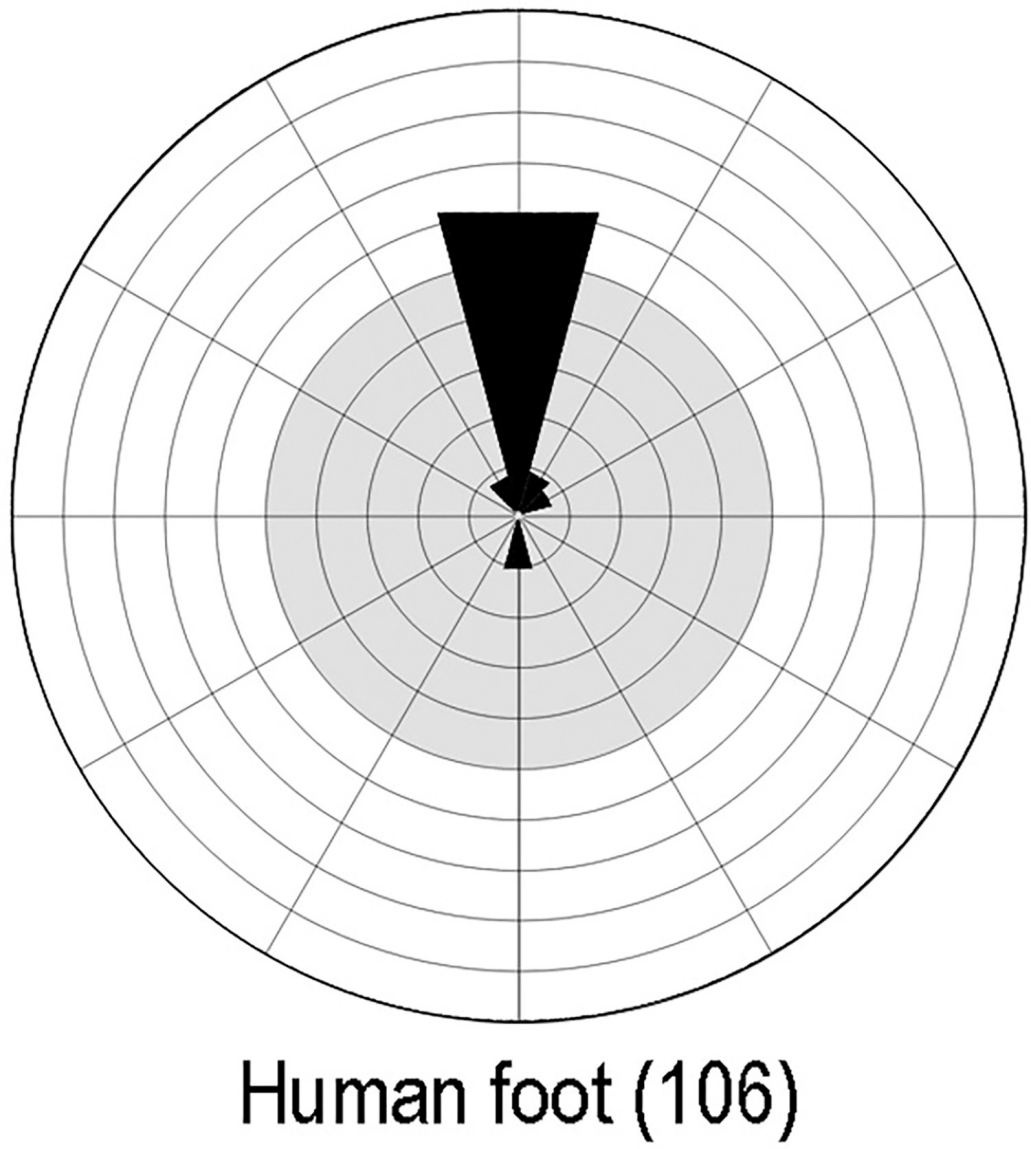
Direction of engraved human footprints. A quantitative illustration of the directions in which engraved human footprints are pointing on the rock panels. We illustrate directions in the form of a wind rose diagram aligned in such a way that each direction shown corresponds to the viewer’s perspective.

In terms of the direction on the rock face in which the feet are pointing, there is a highly evident general preference for footprints ‘walking’ upward (Fig 15), which, as Haynes suggested, we might read as walking away from the observer, while a downward orientation is interpretable as the feet walking towards the observer [16].

We are aided here by classifying the twelve possible directions–on the basis of a clock face–in four main categories of ‘upwards’, ‘right’, ‘downwards’ and ‘left’. According to this scheme, almost 80% of the footprints point upwards, 12% downwards, 7.5% to the right and only 1% to the left. Breaking down direction by the sex identified as pertaining to each footprint shows that women’s footprints tend towards greater directional variety, with a lower predominance of upward direction (Table 11).

**Table 11:** Direction of engraved human footprints.

Principal characteristics	Upwards		To right		Downwards		To left		TOTAL	
	N	%	N	%	N	%	N	%	N	%
Female	22	68.8	3	9.4	6	18.8	1	3.1	32	100.0
Male	62	83.8	5	6.8	7	9.5	-	-	74	100.0
TOTAL	84	79.2	8	7.5	13	12.3	1	0.9	106	100.0

Direction of the engraved human footprints, broken down by sex.

## Discussion

### Evident engraver preferences in the depiction of animal tracks and human footprints

When looking at the engraved rock art of the Doro! nawas, we can distinguish the motifs of humans and animals reasonably clearly due to the absence of their combination in forms such as therianthropes. The depictions of tracks/footprints further include no specimens that show ambiguity as to whether they are of human or animal feet. Our analysis demonstrates that the engravers of these sites endowed both human footprints and animal hooves or paws with an equal depth of information that enables expert analysts to identify specific features such as the sex and age of the individual to whom the tracks or footprints pertain. To the untrained eye, the human engraved footprints could appear more stylised than most of the animal tracks; it was an unexpected outcome that they are an equally rich source of information as the animal tracks. The execution and distribution of these footprints are not distinctly different from those of the animal tracks. It appears to be the case that prehistoric engravers did not produce generic human footprints without markers of sex or age. Similarly, the engravings of animal tracks do not include generic forms; instead, each is specific. This finding is highly noteworthy given the fact that up to 80% of the human figures that appear in the rock paintings of the wider central western Namibian region are ’zero-marked’ [79], that is, in this context, unspecific as to sex and age.

In our view, the data we outline in the preceding sections provide evidence that engravers did not incorporate features into their track depictions at random. In terms of animal tracks, their preference was clearly for particular species–those we have characterised as ‘frequently’ depicted–consisting primarily of quadrupeds (giraffe, white and black rhino, kudu, springbok, bushpig, oryx/gemsbok, ostrich, duiker, leopard, warthog and zebra), but also including at least one bird (guinea fowl). All other species of the total of 39 depicted are ‘less frequently’ or even ‘rarely’ engraved. We note in particular the rich variety of predatory quadruped tracks within our sample (felines: African wildcat, caracal, cheetah, leopard and lion; canines and others: aardvark, aardwolf and jackal); there is no equivalent of this diversity in the depictions of animals in the general body of rock engravings of the area. Among the ‘canines and others’ category, we tend to find insectivores (which to some extent would even be true of jackals) rather than true carnivores such as hyena or wild dog (this said, Scherz identified hyena tracks twice in /Ui//aes-Twyfelfontein [50]).

In terms of the age categories into which the animal tracks and human footprints depicted fall, we observe distinct preferences; among animal tracks, adult animals outnumber juveniles, whereas among human footprints the proportions are inverted. A similar divergence emerges in terms of laterality, with left feet predominating in animal tracks and right feet among human footprints. Tracks of male animals are a little dominant, footprints of male humans clearly so. Only a few animal species (bushpig, duiker and rhino sp.) show the inverse distribution. The artists of the Doro! nawas appear to depict the front legs of quadrupeds more frequently than their hind legs. These various features reveal divergent preferences and priorities in the depiction of animal tracks and human footprints respectively; the rarity of superimpositions and trackways, by contrast, is indicative of the operation of particular conventions that hold for both animal tracks and human footprints. It seems that engravers concentrated on producing isolated, individual forms rather than on representing movement; a focal concern with individual items likewise emerges in the absence of scenes that might show a number of subjects interacting.

On the basis of the analysis thus far, we might observe an overarching pattern underlying these engraved representations. The engravers appear to use the relative direction of the tracks on the rock surface to express, in a notably tangible form a specific characterization of each species. An illustrative example appears in Fig 12, where the outlier status of zebra becomes evident. Duiker is an outlier in a somewhat different way; rather than the erratic distribution we witness in zebra, this species shows the most pronounced preference for the downward direction. Another conspicuous feature of the sample, in terms of track direction, is the emergence of what are fundamentally two clusters of species (Fig 13). A clear tendency towards downward direction occurs among kudu, springbok and duiker, with a less strong trend observable in bushpig and warthog. All of these animals would seem to be good hunting prey; kudu is the only large species among them. Animal tracks typically depicted pointing upward form an even more compact cluster in Fig 13, encompassing large species, some of which can be dangerous to humans, as well as the birds. Any interpretation of these clusters must remain in the realms of speculation; they do, however, bring to mind the unpredictable character of cognitive categories which George Lakoff set out in his seminal study *Women*, *Fire*, *and Dangerous Things* [80].

Haynes analysed the direction of tracks at six sites in Zimbabwe, where, across all animal species, upward direction occurred in 62% of the figures, whereas 32% went downwards [16]; both these figures are in good alignment with our data. One site in Haynes’ study, with an unusual south-facing shelter, represents an outlier, in that 54% of the prints recorded there show a downward direction [16].

### Ecological implications

Among the animals and birds that feature in these rock art sites in the Doro! nawas mountains, we note a number of species that do not occur in the region today because they require a more humid climate and environment than that currently prevailing; these are blue wildebeest, buffalo, bushbuck, bushpig, vervet monkey, roan antelope, red-billed teal, and, to an extent, eland, marabou, red-crested korhaan and open-billed stork [cf. 81–83]. All other animals and birds that appear in the engravings continue to live in the region today (personal communication from Breunig, 09/2018; assertion on the basis of automatic camera surveillance at a waterhole in Doro! nawas). The comparatively large number of animal species that depend on wetter biotopes calls for an explanation and there are two hypotheses regardless of the chronological depth hidden in the depictions: Either the engravings were made by people who had a thorough knowledge of the more humid regions well over 300 km north or east of the sites, or the period during which the tracks were engraved was considerably wetter than today, enabling these species to live in the area. Robust support for either of these hypotheses would call for comparative analysis of the region’s archaeological finds [84] alongside material excavated at sites such as that along the Kavango; this would enable the identification and dating of interactions and migrations between these areas. The fact that archaeological assemblages of the LSA from southern Angola and central Namibia are closely related during the Early Holocene [85] does not in itself constitute reliable evidence for interactions and movements of populations between the Doro! nawas engravers and groups living in regions with more humid climates. Wetter conditions probably prevailed in the region in the period 4200–6000 calBP [86]; however, the extent to which this affected vegetation and fauna remains uninvestigated.

### Comparing the sample with /Ui//aes-Twyfelfontein (central Namibia)

One of the most immediately noteworthy findings of our analysis of tracks in the rock engravings of the Doro! nawas region is the relative abundance of human footprints (20%, a figure corroborated by the dataset for the same region in Fels [87]). Regarded on a larger scale, this art tradition typically incorporates depictions of human figures [50] at a very low frequency, of between 0.5% [52, 54] and 1.8% [87] of all depictions. Taking the human footprints as representing human figures, we note that even this proportion, of 20% of the depictions, is still far below the approximately 80% of figures representing humans in the rock paintings of central Namibia [52, 79, 88]. This finding is therefore distinct in character and, in our view, constitutes a strong indication that engravings of tracks may occupy a particular position or a specific field of meaning within the epistemic field of engraved art. Other facts point similarly in this direction: the variety of species is much richer in the tracks analysed in the present study than in all other investigations into animal motifs in the region, and in particular at /Ui//aes-Twyfelfontein (Table 12) [50, 52, 54]. Further, the engravings of animal tracks examined in this study are generally true to nature, with very few stylisations (only two tracks of rhino, two of kudu, and one each of leopard, African wildcat and zebra are stylised) and no hybridisations, such as merging of different animals and therianthropic motifs [52]. Other notable absences from the present study’s sample are tracks of domestic animals and of reptiles. Kinahan & Kinahan [52] identified 5% of the animals they investigated at Ui//aes-Twyfelfontein as cattle, but these animals did not appear to be represented in tracks [cf. 50 Tafel 115/2, 52]; reptiles generally do not appear in rock engravings of the region.

**Table 12:** Comparative summary of animal track engravings.

Species	Animal tracks												Full animal depictions		
	Doro! nawas								Twyfelfontein*		NW Namibia**		Twyfelfontein***	NW Namibia**	
	RAS-6-B N	RAS-6-C	RAS-8-H	RAS-8-N/NE	RAS-8-N/SW	RAS-8-O	TOTAL								
	N	N	N	N	N	N	N	%	N	%	N	%	%	N	%
Aardvark	-	-	-	-	-	1	1	0.2	1	0.3	1	0.1	-	4	0.2
Aardwolf	-	-	-	-	-	1	1	0.2	-	-	-	-	-	-	-
African wildcat	-	1	-	1	1	1	4	0.8	-	-	-	-	-	1	0.04
Baboon	2	1	-	-	-	3	6	1.2	1	0.3	5	0.3	-	8	0.3
Black stork	-	-	-	-	-	1	1	0.2	-	-	-	-	-	-	-
Blue wildebeest	-	1	-	-	2	5	8	1.6	9	2.7	136	8.3	-	27	1.1
Buffalo	3	-	-	2	-	2	7	1.4	-	-	-	-	-	-	-
Bushbuck	-	-	-	-	-	4	4	0.8	-	-	-	-	-	-	-
Bushpig	1	-	-	15	2	-	18	3.5	-	-	-	-	-	-	-
Caracal	1	2	-	-	1	-	4	0.8	-	-	3	0.2	-	-	-
Cheetah	-	-	-	-	-	1	1	0.2	-	-	-	-	-	-	-
Duiker	1	2	-	4	2	4	12	2.5	-	-	-	-	-	-	-
Eland	-	-	-	-	-	2	2	0.4	12	3.6	156	9.5	-	37	1.5
Elephant	-	2	-	-	1	3	6	1.2	7	2.1	22	1.3	-	107	4.5
Francolin	-	-	-	1	-	-	1	0.2	-	-	-	-	-	1	0.04
Giraffe	18	3	-	11	7	42	81	15.8	62	18.8	296	18.0	40	814	34.0
Guinea fowl	1	5	10	-	-	7	23	4.5	-	-	-	-	-	6	0.3
Human (foot)	12	22	1	-	1	70	106	20.3	-	-	370	22.5	-	111	4.6
Jackal	-	-	-	-	-	1	1	0.4	-	-	-	-	-	18	0.8
Kori bustard	6	2	-	-	-	-	8	1.6	24	7.3	26	1.6	-	7	0.3
Kudu	2	2	-	8	2	22	36	7	37	11.2	84	5.1	-	40	1.7
Leopard	1	5	-	-	-	7	13	2.5	18	5.5	55	3.3	-	12	0.5
Lion	1	2	-	-	-	5	8	1.6	22	6.7	82	5.0	-	10	0.4
Marabou	-	-	2	-	-	-	2	0.4	-	-	-	-	-	-	-
Monkey	-	-	-	-	1	3	4	0.8	-	-	-	-	-	-	-
Open-billed stork	-	-	-	-	-	1	1	0.2	-	-	-	-	-	-	-
Oryx	-	6	1	-	-	11	18	3.5	35	10.6	125	7.6	8	170	7.1
Ostrich	2	4	-	-	1	11	18	3.5	18	5.5	34	2.1	6	395	16.5
Porcupine	-	-	2	-	-	-	2	0.4	-	-	-	-	-	1	0.04
Rabbit	-	-	-	-	-	2	2	0.4	-	-	-	-	-	1	0.04
Red-billed teal	-	-	-	1	-	-	1	0.2	-	-	-	-	-	-	-
Red-crested korhaan	2	3	-	-	-	1	6	1.2	-	-	-	-	-	-	-
Rhino sp.	-	-	-	-	-	4	4	0.6	42	12.7	82	5.0	19	282	11.8
Rhino, black	5	8	-	-	1	7	21	4.3	-	-	-	-	-	-	-
Rhino, white	5	4	-	-	3	9	21	4.1	-	-	-	-	-	-	-
Roan antelope	-	-	-	-	-	3	3	0.6	-	-	-	-	-	-	-
Secretary bird	-	-	-	-	-	1	1	0.2	-	-	-	-	-	-	-
Springbok	-	7	1	4	2	14	28	5.5	17	5.2	34	2.1	-	40	1.7
Steenbok	-	-	-	-	-	3	3	0.6	-	-	-	-	-	-	-
Warthog	1	-	-	5	1	6	13	2.5	3	0.9	37	2.3	-	15	0.6
Zebra	1	6	-	1	-	4	12	2.3	22	6.7	95	5.8	12	290	12.1
TOTAL	65	88	17	53	28	262	513	100.0	330	100.0	1643	100.0	85	2397	100.0

Synoptic table of animal and track engravings in the area of the present research and neighbouring regions *[50], **[50 Liste 5c], ***[52]

The comparative list of motifs in previous studies (Table 12 and Figs 9–11) shows that all species that Scherz identified by their tracks in Twyfelfontein are also present in the neighbouring Doro! nawas sample (with the exception of hyena), while 20 additional species occur at Doro! nawas.

The analysis of preferences for left or right feet does not provide a consistent pattern. Ethnographic observation among the San has made the field aware that the right foreleg of eland was associated with boys’ initiation and the left with girls’ initiation [89]; this information, however, does not help us in our understanding of the Doro! nawas engravings as a whole.

### Comparing the sample with other southern African rock art traditions

Looking beyond the central Western Namibian region of rock art, we can take the Tshikongomoti shelter in the Limpopo basin, South Africa, as a case for comparison [5] with Doro! nawas’ RAS 8-O. The authors of a study on this site presumed that it could be “the largest site containing track engravings in South Africa” [5], given the more than 90 engraved and painted tracks it encompasses. The species a professional hunter identified at Tshikongomoti are elephant, hippopotamus, giraffe, zebra, kudu, wildebeest, tsessebe, hartebeest, bushbuck, impala, waterbuck, eland, sable antelope, and warthog (the authors provide no information as to whether this sequence reflects the order of the species’ frequency; it would not seem so judging from the small graphical reproduction of the panel the publication includes [5]). Although this list contains the larger herbivores found in that environment, and to this extent allows comparability to the Doro! nawas track engravings, we note a striking divergence, in that the South African tracks do not include any from carnivores or birds. Another South African site with numerous tracks is Kinderdam in the Karroo, which features 71 human footprints, but only two animal tracks [90]. The authors do not go into detail about these human footprints.

Engravings of animal tracks occur in several areas of Zimbabwe, with most of them concentrated in Hwange National Park in the west of the country [91]. Across all sites analysed in this study, the author identified 14 animal species, with zebra being most frequent (56 occurrences), followed by kudu and giraffe; other animals in this sample are (in descending order of frequency) birds, quagga, buffalo, baboon, felines, elephant, crocodile, eland, warthog and impala; human footprints occur twelve times [91]. Of a total of 40 species identified at the Bumbusi site in Hwange National Park, zebra are most frequently depicted, followed by impala and kudu [16]. Interestingly, most tracks at this site, except those of zebra, point downwards on the rock walls.

Walker compared several sites featuring track engravings in Botswana and Zimbabwe. At two of the Botswanan sites, he found human footprints to amount to around 20% of the engravings, while at the other four sites they were absent or very rare [22]. At the sites with human footprints, the tracks of herbivores were either absent or their frequency was below 5%. At sites without human footprints, the tracks of herbivores predominated, with frequencies of between 60% and 90%; the second largest category was tracks of felines while bird tracks were absent.

None of these sites we have cited for comparison shows a frequency pattern similar to that observable in the Doro! nawas mountains. This would point to a variety of meanings attributable to, and each distinct in the cited bodies of art, a supposition which would be in line with the polysemy of track engravings as postulated by Eisenberg-Degen and Nash [92].

A feature of track depictions that already occurs in Palaeolithic art is the placement of a track sign in the depiction of an animal in place of the paw or hoof (an example is the elephant on the panel of lions in Grotte Chauvet [93]). There are some renowned examples of this kind in /Ui//aes-Twyfelfontein, but we noted none on the panels investigated for the present study.

### Generalised expectations regarding the meaning of track engravings derived from the Doro! nawas data

We have observed that, instead of producing more or less generic tracks such as those found in today’s game guidebooks and tracking guides, the engravers who worked at the sites we studied expressed their specialist knowledge in every depiction they made. They evidently had clear ideas of how a particular species should be represented in its track. Looking first at the direction of tracks—a feature detectable even by a non-specialist—our data demonstrate that only zebra has the directions of their tracks represented apparently at random, while all other animals show clear, yet different biases in the directions depicted. In light of features such as this and the diversity of species engraved, the question arises as to the purposes for which these engravings were produced. We will now attempt to develop an understanding of what these various specificities might mean in the context of the engravings, and therefore of rock art of this type more generally, by setting out some hypothetical expectations around rock art of this type and assessing the extent to which the data bear them out.

Expectation 1: All features of the engravings as analysed in our study are equally meaningful and therefore they are randomly distributed across all depictions.

Comment: The data do not support Expectation 1; the features the engravers include in their depictions are far from random in character, with each species evincing a relatively specific choice of favoured features. Even the seemingly random orientations of zebra tracks confer a specificity of meaning on their depictions due to the outlier status of this lack of clear bias.

Expectation 2: Tracks mainly serve to represent animals with particular significance to hunters, in that they provide the best meat or considerable quantities of fat. If maximising the rate of return from a hunt was the key argument for choosing to hunt a particular species or a particular sex of animal within a species, this may entail a preference for adult males, as favoured prey, in the depictions.

Comment: We do not find convincing support for Expectation 2 in the data. Adult males predominate drastically in zebra, and, to a lesser extent, oryx. The Ju/’hoansi hunters maintain that zebra (likewise ostrich) are not edible, although Hei//om San do hunt and eat them (personal communication from Jan Tsumeb, 2019). The engravings include many other animals, such as rhino and felines, that were not typical prey for hunter-gatherers of the period because hunting them was too hazardous an enterprise.

Expectation 3: Tracks of an animal are central to hunting in the Kalahari, as they lead the hunter to their prey by means of tracking [35]. This is in line with the observation by Tsamgao Ciqae (personal communication, 2013) on representations of tracks having a higher significance in rock art than do depictions of the animals to which they pertain.

Comment: The observation we cite in Expectation 3 represents a positivist view of rock art, expressed by a single indigenous tracking expert and hunter who perceives it to constitute actual, sensorily perceptible and verifiable evidence. Notwithstanding the subjective character of this assertion, it is nevertheless instructive in warning Western scientific disciplines and assumptions against underestimating the significance of tracks in prehistoric art. But as an individual statement this does not suffice to allow generalisations for the meaning of rock art.

Expectation 4: Given the currently favoured view in research into rock art, specifically into that of southern Africa, that this art is fundamentally shamanistic in character and purpose [see, for example, 94, 95], we might expect, if this view were accurate, that such art would tend towards depicting rather small numbers of animal species, of which a single or a very few species (animals associated with power, particularly eland) would stand out markedly and appear widely distributed across all sites [96].

Comment: The high frequency of giraffe tracks among these engravings, with giraffe appearing on all but one panel, meets Expectation 4 in part. The predominance of giraffe likewise holds true in the animal engravings [50, 52] and even in the rock paintings of the wider region, particularly in the Dâureb/Brandberg, where giraffe is among the most numerous animal motifs [79]. Giraffe tracks constitute 20% of the animal track engravings in the study’s sample. The next most frequently depicted animals are the two species of rhino, amounting together to 11%, followed by kudu (9%) and springbok (7%; in the rock paintings of Dâureb/Brandberg they are the most numerous species, representing more than 30% of all animals depicted [79]). The dominant species thus appears about twice as frequently as other relatively frequently depicted species, a finding in line with the postulate by Sauvet et al. in reference to a shamanistic context [96]. However, if we understand the two basic techniques of prehistoric art, engraving and painting, as emerging from a culture whose practice of shamanism was homogeneous to some extent [53, 94], then the differences in the predominant animals in paintings and engravings respectively render a shamanistic context doubtful. Further, eland, which play a crucial role in the shamanism hypothesis [94], are extremely rare in Namibian prehistoric art in general, and the large number of human footprints observable would be inconsistent with a shamanism hypothesis. It would be inaccurate to consider the footprints as depictions of shamans’ feet due to the preponderance of juveniles–even babies–over adults among the engravings.

The sample of the present study is much too small, in terms of number of sites, to supply a reliable assessment of distribution patterns across the landscape. A robust analysis would depend on the accessibility of the entire region’s body of rock art [87].

Expectation 5: Following Sauvet et al., we could put forward an alternate interpretation of the track engravings as having a totemic function, under the assumption that the track of an animal represents *pars pro toto* of the totem animal; according to these authors, a partial indication of totemic art is evident from each motif appearing only at certain sites, with all motifs occurring at low but similar frequencies [96].

Comment: Even given the limited scope of the research described in this study, it is evident that these patterns of distribution are barely applicable to the art of the Doro! nawas region, refuting this expectation.

Expectation 6: A third possible function of art suggested by Sauvet et al. is secular (with a very broad spectrum of mundane manifestations); in this case, the same motifs would be present across almost all sites, with all motifs occurring at low but similar frequencies [96].

Comment: The same applies to this hypothesis as to Expectation 5, in that even this limited sample indicates that this type of distribution does not apply here.

Expectation 7: The indigenous tracking experts put forward the view that the track engravings could have served to teach newcomers. If this were the case, a wide variety of species and features would be useful in terms of illustrating subtle differences. This is also the hypothesis espoused by the indigenous San trackers who assisted Nankela in identifying engraved animal tracks in the Namibian Erongo mountains [97].

Comment: Expectation 7 appears met in both the variety of species depicted and the breadth of features used in the engravings. The inclusion of human footprints would serve a useful purpose in this context, as they require study as much as animal tracks do. This said, if the engravings were indeed elements of a comprehensive instructional scheme, we might expect the features depicted to cover the entire range for each species; that is, they would include young and old, male and female specimens, alongside the depiction of all four feet of quadrupeds in roughly equal proportions–which, as our findings indicate, is certainly not the case. The study of tracks using these engravings as models would leave initiates deficient in particular aspects of their knowledge. Further, this form of learning in a “classroom” environment would be atypical of hunter-gatherer practices [43]; inside the RAS 8-N crevice it would indeed be impossible. A further argument against this “educational” hypothesis is the fact that it would make little sense to concentrate, in this poorly lit place, the tracks of a single species which did not even occur in this area (assuming that the present arid conditions prevailed at that time). This hypothesis further fails to explain the systematic differences in the directions of the tracks, since no utility for the learning of tracking could emerge from the directions as shown on the rock walls, so that we might instead have expected random directions or the use of one direction only.

Expectation 8: The tracks are intended to represent the same animals that this rock art tradition depicts in full silhouette, i.e. fundamentally large herbivores (that were preferred prey for hunting, even if success in such endeavours was rare) [cf. 42].

Comment: Our data do not meet Expectation 8. The tracks identified in the present study represent 39 species; by contrast, even Scherz, who provides the most detailed count of species in Twyfelfontein,–distinguished only 19. The “surplus” species that appear in the tracks analysed for the present study are either rather small animals or typical of more humid environments, and include several species of birds that are rare in terms of the frequency of their occurrence in rock art in general. It is only once we look at a study of the wider regional range, including the entire northwest of Namibia as far as the Kavango River, that the number of species occurring in animal motifs is larger than the number of species represented in tracks [50]. Scherz’s findings relied exclusively on his own knowledge of animal tracks. For this entire region, Scherz identified 37 species in total, 19 of them in tracks and animal depictions and four exclusively by their tracks. This means that a number of species in the present study were identified only by the indigenous tracking experts and are not listed by Scherz notwithstanding the possibility that Scherz’s category “Buck/animal” may include ungulates which Scherz did not recognise but which the trackers were able to identify in Doro! nawas mountains: buffalo, bushbuck, duiker, roan antelope and steenbok. Analogously to our experts’ identification of the tracks of “rarely” depicted animals, which occur only once or twice, Scherz identified eleven species in animal depictions (not tracks) that appear at this low frequency across the whole of north-western Namibia, among them animals such as penguin and pangolin [50].

The discussion around these various hypotheses on the art’s purpose and meaning shows that the shamanism hypothesis has some more bearing than others, yet, as we have seen, is only tenable if we disregard the high frequency of human footprints, that are not consistent with the role of animals in a trance context [e.g., 94]. Further, the rich diversity of species in this sample, a third of which appear only once or twice, is not in line with the depiction of few species, replete with meaning and potency, which research regards as key identifying features of shamanistic art [96]. The “rarely” depicted animals seem rather to send an ambiguous and idiosyncratic message. Further, an emphasis on shamanism in interpreting this art would provide no explanation for the variety of animals depicted and for the clear distinctions the engravings make among the species’ various features. In other words, it appears that there must be some specific information encoded in these tracks and articulated through specialised ichnological knowledge. The features investigated in this study, taken together, point to the tracks being endowed with complex meanings; attaining even an approximation of these would require researchers to call upon ethnographic data and indigenous knowledge–a necessity of which future research would do well to take heed.

## Conclusion

This study, in its examination of engraved animal tracks and human footprints in prehistoric hunter-gatherer rock art analysed by indigenous tracking experts, shows that engravers made evident and deliberate choices around the types of tracks of a particular animal they would most frequently depict. These preferences confer a specific character upon each species featured, both through the preferred direction of the tracks on the rock wall and via features such as a preference for young males among species such as bushpig and duiker or for older females in, for instance, leopard and guinea fowl.

Among the various hypotheses we have outlined around the tracks’ significance, none proves convincing, due in part to the significant proportion of human footprints in this body of track engravings. Were we to disregard the human footprints for these purposes, the shamanism hypothesis in particular could gain explicatory power, but there is no obvious reason for the human footprints’ exclusion except their weakening of this hypothesis. The same type of complex information that tracks in general convey to the experienced tracker may reside likewise in elaborate engravings of the entire animals, i.e., there may be encoded information about behaviour, for example; confirmation of this would require systematic investigation, that may usefully involve indigenous tracking experts.

**Fig 16:**
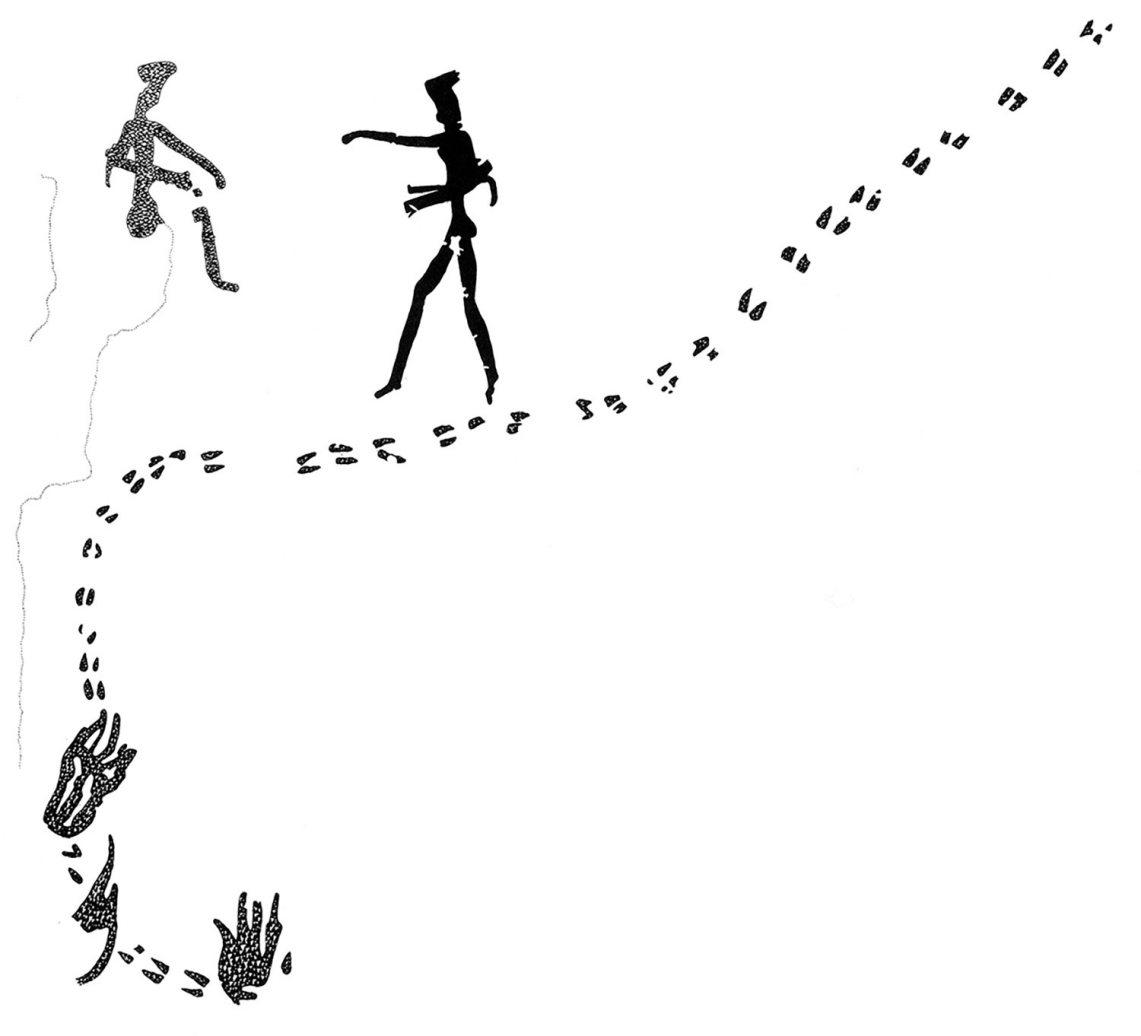
Painted animal tracks in rock art in Namibia. In the rock paintings of Namibia, animal tracks appear less frequently than in engravings, but where they do occur–as in this example from Daûreb/Brandberg (site I 109, [98])–they form trackways. This reproduction of the panel shows all depictions at this site; it appears, then, as if the animal which left the tracks had never been painted. The pictorial elements from which the tracks issue could be bushes. The two human figures, although they are carrying hunting equipment under their arms, seem to be occupied more with themselves than with the animal tracks.

With the exception of guinea fowl, the sample contains no trackways that convey information on where an individual animal was moving to or from, vital detail in real-life tracking. By contrast, the few rock paintings at the nearby Dâureb/Brandberg which feature animal tracks depict them as long trackways (Fig 16). It is barely conceivable that the depiction of a track’s direction on the rock wall (upward, downward etc.) compensates for the absence of this significant piece of information. Any speculation on the significance of the directions shown in the engravings, such as their potential signification of tracks coming from or leading to other worlds, cannot suffice as a complete explanation for the meanings encoded in this engraved art.

An attempt to attribute any definite meaning to the track engravings of the Doro! nawas mountains must remain guesswork and speculation in view of the rich polysemy of tracks [92] that occur worldwide. For the time being we can maintain that the track engravings from the prehistoric hunter-gatherer culture(s) that existed in Namibia appear to have had an epistemic purpose that rested on thorough positivist, empirical knowledge of the lifeworld from which they emerged. Whatever the deeper and symbolic meanings of these engravings, it could only emerge in its entirety through a direct conversation with the artists. It may be that further statistical analysis centring on other, as yet uninvestigated features of the engraved tracks could enable researchers in this field to identify some cognitive groupings of the animals depicted which are neither self-evident nor self-explanatory–both of which are general characteristics of cognitive categorisations from a global point of view [80]. The difficulty we note here is one that arises in the interpretation of all prehistoric art. Consulting present-day indigenous experts can partly mitigate this issue, enabling Western researchers access to greater depth of insight through the outstanding precision and plausibility of indigenous knowledge; yet often, as in this case, the precise meaning and context of the art will remain elusive.

### Ethical standards

The research that took place in this project draws heavily on the knowledge of the Ju/’hoansi people from the Nyae Nyae region in north-eastern Namibia. Our observation of the ethical standards that are crucial to such work was guided by the San Code of Research Ethics [99]. On this basis, and following on from the experience of collaborative research stemming from the Tracking in Caves project (2013), the research team developed an ethics protocol with recommendations for archaeological research [100].

## Supporting information

S1 Checklist Inclusivity in global research. Additional information regarding the ethical, cultural, and scientific considerations specific to inclusivity in global research.
(PDF)
